# Differential Roles of TREM2+ Microglia in Anterograde and Retrograde Axonal Injury Models

**DOI:** 10.3389/fncel.2020.567404

**Published:** 2020-11-20

**Authors:** Gemma Manich, Ariadna Regina Gómez-López, Beatriz Almolda, Nàdia Villacampa, Mireia Recasens, Kalpana Shrivastava, Berta González, Bernardo Castellano

**Affiliations:** Department of Cell Biology, Physiology, and Immunology, Institute of Neuroscience, Universitat Autònoma De Barcelona, Barcelona, Spain

**Keywords:** IL-10 (interleukin-10), IL-6 (interleukin-6), phagocytosis, proliferation, neuroinflammation, microglial clusters, P2RY12, APOE

## Abstract

Microglia are the main immune cells of the central nervous system (CNS), and they are devoted to the active surveillance of the CNS during homeostasis and disease. In the last years, the microglial receptor Triggering Receptor Expressed on Myeloid cells-2 (TREM2) has been defined to mediate several microglial functions, including phagocytosis, survival, proliferation, and migration, and to be a key regulator of a new common microglial signature induced under neurodegenerative conditions and aging, also known as disease-associated microglia (DAM). Although microglial TREM2 has been mainly studied in chronic neurodegenerative diseases, few studies address its regulation and functions in acute inflammatory injuries. In this context, the present work aims to study the regulation of TREM2 and its functions after reparative axonal injuries, using two-well established animal models of anterograde and retrograde neuronal degeneration: the perforant pathway transection (PPT) and the facial nerve axotomy (FNA). Our results indicate the appearance of a subpopulation of microglia expressing TREM2 after both anterograde and retrograde axonal injury. TREM2+ microglia were not directly related to proliferation, instead, they were associated with specific recognition and/or phagocytosis of myelin and degenerating neurons, as assessed by immunohistochemistry and flow cytometry. Characterization of TREM2+ microglia showed expression of CD16/32, CD68, and occasional Galectin-3. However, specific singularities within each model were observed in P2RY12 expression, which was only downregulated after PPT, and in ApoE, where *de novo* expression was detected only in TREM2+ microglia after FNA. Finally, we report that the pro-inflammatory or anti-inflammatory cytokine microenvironment, which may affect phagocytosis, did not directly modify the induction of TREM2+ subpopulation in any injury model, although it changed TREM2 levels due to modification of the microglial activation pattern. In conclusion, we describe a unique TREM2+ microglial subpopulation induced after axonal injury, which is directly associated with phagocytosis of specific cell remnants and show different phenotypes, depending on the microglial activation status and the degree of tissue injury.

**Graphical Abstract:**
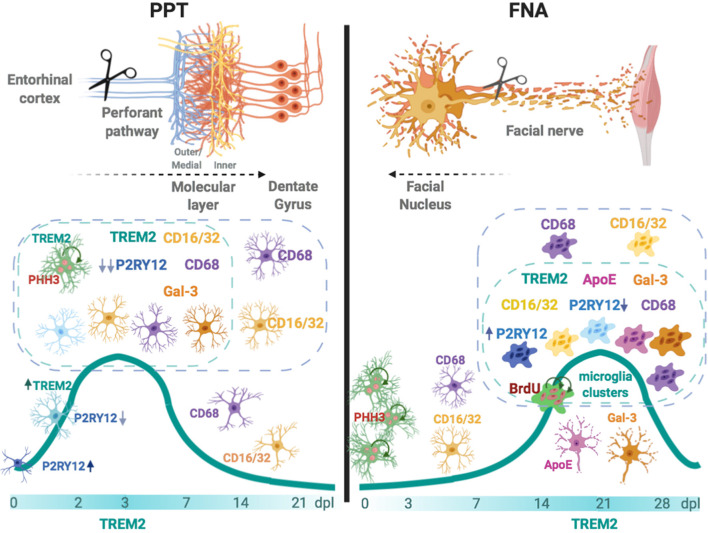
In the left, after an anterograde axonal injury such as the perforant pathway transection (PPT), TREM2 is rapidly upregulated in a subpopulation of microglia located in the molecular layer. TREM2+ microglia, which can be proliferative, show co-localization with phagocytic CD68 and CD16/32, occasionally with TREM2 phagocytic ligand Galectin-3 (Gal-3), and demonstrates an important P2RY12 downregulation. In the right, after facial nerve axotomy (FNA)—a retrograde axonal injury—TREM2+ microglia is upregulated at late time-points, coinciding with the formation of phagocytic clustered microglia. Microglia clusters can be CD68+ and CD16/32+, may show expression of TREM2 ligands ApoE and Galectin-3, both molecules also observed in the surface of facial motor neurons, and can express variable levels of P2RY12. Overall, our results suggest that TREM2+ microglia is a subpopulation that phagocytes more efficiently specific cell remnants, mainly myelin and degenerating neurons, and its expression prepares the way for injury repair.

## Introduction

Microglial cells are the principal myeloid cells within the central nervous system (CNS), which play an immune surveillance function under homeostasis and after damage or pathological insult (Colonna and Butovsky, 2017; Wolf et al., 2017). A molecule that gained attention in the last years by its involvement in the modulation of microglia is the Triggering Receptor Expressed on Myeloid cells 2 (TREM2), an innate transmembrane type I receptor of the immunoglobulin superfamily that is expressed only by myeloid cells (Colonna, 2003). Several studies have strongly linked variants in the TREM2 gene with increased risk of developing Alzheimer’s disease (AD), fronto-temporal dementia, and Nasu-Hakola disease and, to a lesser extent, Parkinson’s disease and amyotrophic lateral sclerosis (reviewed in Jay et al., 2017b).

In the brain, TREM2 is exclusively expressed by microglia, with variations depending on the CNS region (Schmid et al., 2002), and is generally upregulated *in vivo* upon inflammatory conditions or in aging (Gratuze et al., 2018). A plethora of ligands bind to TREM2, including anionic ligands, such as phospholipids or sulfatides, lipoproteins like ApoE, β-amyloid, and also DNA (reviewed in Kober and Brett, 2017). Upon ligand binding, TREM2 interacts with DAP12 and results in a wide range of functions, including proliferation, migration, pro-survival signal, lipid sensing, phagocytosis, and energy metabolism (reviewed in Painter et al., 2015; Jay et al., 2017b), mainly aimed at containing and removing apoptotic or degenerated cells produced during neuronal damage (Takahashi et al., 2005, 2007; Hsieh et al., 2009; Krasemann et al., 2017; Deczkowska et al., 2018). Recently, single-cell RNA-sequencing analysis in the CNS tissue linked TREM2 with the differentiation of a newly identified specific microglial subtype appearing in mice in neurodegenerative conditions and aging, the so-called disease-associated microglia (DAM; Keren-Shaul et al., 2017; Deczkowska et al., 2018) or microglia associated to neurodegeneration (Krasemann et al., 2017). These microglia play a key role in chronic neurodegenerative conditions and show a unique transcriptional and functional signature highly differing from homeostatic microglia, characterized by the overexpression of other genes, such as *Clec7a, Lgals3, ApoE*, and the downregulation of microglial homeostatic genes, such as *P2ry12* or *Tmem119*.

The role of TREM2 has been mainly reported in chronic experimental models of AD and other chronic disorders—like experimental autoimmune encephalomyelitis (EAE, reviewed in Karanfilian et al., 2020)—and, to a lesser extent, in different acute neuroinflammatory conditions, including cuprizone-induced demyelination, stroke, and traumatic brain injury, among others (reviewed in Deming et al., 2018). However, little is known about its expression after axonal CNS lesions, and even less about the role of TREM2 in those lesions that are associated with a reparative process. Indeed, studies on microglial TREM2 function after spinal nerve injury (Kobayashi et al., 2016) showed an upregulation of TREM2, which was also detected in the microglial transcriptome after facial nerve injury (Tay et al., 2018). Moreover, in these and other studies based on TREM2KO and DAP12KO mice, reduced secretion of pro-inflammatory cytokines and a lower neuronal death rate were reported after axotomy (Kobayashi et al., 2015; Krasemann et al., 2017), pointing towards an important role of TREM2 in the modulation of the inflammatory response and the resolution of nerve injury (Kobayashi et al., 2016).

In this context, the main objective of our work is to study TREM2 expression and its function in two well-established models of anterograde and retrograde axonal degeneration —with an associated reparative process—: the perforant pathway transection (PPT: transection of the principal pathway that connects the entorhinal cortex with the hippocampus) and the facial nerve axotomy (FNA), respectively. Also, we explored whether changes in microglia activation by local pro-inflammatory or anti-inflammatory microenvironments, using transgenic mice with IL-6 or IL-10 overproduction (Almolda et al., 2014; Villacampa et al., 2015; Recasens et al., 2019), are able to modulate TREM2 expression after PPT and FNA.

## Materials and Methods

### Animals

GFAP-IL6 transgenic (Tg) mice, GFAP-IL10Tg, and their corresponding wild-type (WT) C57BL/6 littermates from both sexes were used in this study. GFAP-IL6Tg was constructed and initially studied by Prof. Iain Campbell (Campbell et al., 1993), while GFAP-IL10Tg has been constructed and characterized by our laboratory (Almolda et al., 2015). All mice were housed and bred in the Institute of Neurosciences of the *Universitat Autònoma de Barcelona* under a 12 h light/dark cycle, with food and water *ad libitum*. All experimental animal work was conducted according to Spanish regulations (Ley 32/2007, Real Decreto 1201/2005, Ley 9/2003 and Real Decreto 178/2004) in agreement with European Union directives (86/609/CEE, 91/628/CEE, and 92/65/CEE) and was approved by the Ethical Committee of the Autonomous University of Barcelona. Every effort was made to minimize the number of animals used to produce reliable scientific data, as well as animal suffering and pain.

### Perforant Pathway Transection and Experimental Groups

In the first part of the study, 84 WT animals were used, and in the second part of this study, performed with Tg animals, 27 GFAP-IL6Tg, 45 GFAP-IL10Tg, and a total of 72 corresponding WT mice littermates were used. Animals were subjected to wire-knife unilateral PPT, as already described in Jensen et al. (1999). Briefly, animals were anesthetized with an intraperitoneal injection of a solution of ketamine (80 mg/kg) and xylazine (20 mg/kg) at the dose of 0.01 ml/g body weight. Anesthetized mice were placed in a stereotaxic device (Kopf Instruments^®^) and a small window in the skull was created by drilling made in the left side of the skull (4.6 mm dorsal to Bregma and 2.5 mm laterally). A folded wire-knife (McHugh Milleux, m121) was inserted at an angle of 15° anterior and 10° lateral. The knife was unfolded at 3.6 mm ventrally and the perforant pathway—formed by fibers from neurons placed in layers II and III from the entorhinal cortex projecting to the hippocampus—was transected retracting the knife 3.3 mm. Finally, the knife was folded and removed from the brain. After surgery, the skin was sutured with 2–0 silk and the wound was cleaned with iodine. Non-lesioned (NL) and lesioned animals were distributed in different experimental groups and euthanized at 2, 3, 7, 14, and 21 days post-lesion (dpl).

### Facial Nerve Axotomy and Experimental Groups

In the first part of this work, 110 WT animals were used, and for the second part of this study, specifically performed with Tg animals, 32 GFAP-IL6Tg, 32 GFAP-IL10Tg, and a total of 64 corresponding WT mice littermates were used. Animals were anesthetized with a solution of ketamine (80 mg/kg) and xylazine (20 mg/kg) injected intraperitoneally at a dose of 0.01 ml/g and underwent a unilateral FNA as described in Almolda et al. (2014). The skin behind the right ear was shaved and cleansed with 70% ethanol. A small incision was made in the skin and both the trapezius and the anterior digastric muscles were gently separated to expose the right facial nerve. One millimeter of the facial nerve main branch was resected at the level of the stylomastoid foramen. Following the surgery, the skin was sutured with 5–0 nylon. Corneal dehydration was prevented by the application of Lacri-lube^®^ eye ointment. After anesthesia recovery, the complete whisker paralysis was assessed to ensure that complete facial nerve resection was achieved. NL and axotomized animals were distributed in different experimental groups and euthanized at 3, 7, 14, 21, and 28 dpl.

### 5’ Bromodeoxyuridine Injections

To determine microglia proliferation in the FNA model, proliferative cells were labeled with 5’ bromodeoxyuridine (BrdU). BrdU is a synthetic thymidine analog that incorporates into the DNA of dividing cells during the S-phase and can be transferred to daughter cells upon replication. Lesioned WT (*n* = 4) animals were intraperitoneally injected with BrdU (100 mg/kg) diluted in 0.1 M PBS (pH 7.4) every 24 h, from the day of the lesion to 14 dpl, to be sacrificed afterward.

### Tissue Processing for Histological Analysis

Animals were deeply anesthetized with a solution of ketamine (80 mg/kg) and xylazine (20 mg/kg) injected intraperitoneally at a dose of 0.015 ml/g and perfused intracardially with 4% paraformaldehyde in 0.1 M phosphate buffer (pH 7.4). Brains were removed and post-fixed in the same fixative for 4 h at 4°C and, after rinsing in phosphate buffer, cryopreserved for 48 h in a 30% sucrose solution and subsequently frozen in ice-cold 2-methyl butane solution (320404, Sigma–Aldrich). Parallel free-floating coronal sections (30-μm-thick) of the brainstem containing the facial nucleus (FN), in the case of the FNA model, as well as parallel free-floating transversal sections (30-μm-thick) of the brain containing the hippocampus, for the PPT model, were obtained using a CM3050s Leica cryostat, and sections were stored at −20°C in Olmos antifreeze solution until their use. Each experimental group was formed by three to five animals.

### Single Immunohistochemistry

Free-floating cryostat sections were processed for the visualization of TREM2, CD16/32, CD68, and phospho-Histone 3 (pHH3). Briefly, after 10 min of endogenous peroxidase blocking with 2% H_2_O_2_ in 70% methanol and later washes with Tris-buffered saline (TBS, pH 7.4), sections were blocked for 1 h in either blocking solution 1 (BS1), containing 0.2% gelatin (powder food grade, 1.04078, Merck) in TBS with 0.5% Triton X-100 (TBS-T0.5%)—in the case of TREM2 staining—or in blocking solution 2 (BS2), containing 10% fetal bovine serum (FBS) in TBS with 1% Triton X-100 (TBS-T1%)—for CD16/32, CD68 and pHH3–. Then, sections were incubated with sheep anti-TREM2, rat anti-CD16/32, rat anti-CD68, or rabbit anti-pHH3 antibodies (Table 1). Incubations were performed overnight (ON) at 4°C plus 1 h at room temperature (RT) in the case of CD16/32, CD68, and pHH3, or 48 h at RT in the case of TREM2. Sections incubated in media lacking the primary antibody were used as negative control and spleen sections as a positive control. After washes with either TBS-T0.5% or TBS-T1%, sections were incubated at RT for 1 h with the corresponding biotinylated secondary antibodies, followed by 1 h at RT with horseradish peroxidase (HRP)-conjugated streptavidin, diluted in the corresponding BS (Table 1). After washes with TBS, the final reaction was visualized by incubating sections with a DAB kit (SK-4100; Vector Laboratories Incorporation, Burlingame, CA, USA) following the manufacturer’s instructions. Finally, sections were mounted on gelatin-coated slides, counterstained with toluidine blue if considered appropriate, dehydrated in graded alcohols, and, after xylene treatment, coverslipped with DPX.

**Table 1:** List of antibodies and reagents used in immunohistochemistry (IHC).

	Target antigen	Host/conjugation	Dilution	Catalog number	Manufacturer
Primary antibodies	ApoE	Goat	1:2,500	AB947	EMD Millipore
	BrdU	Rat	1:120	Ab6326	Abcam
	CD11b	Rat	1:1,000	MCA74G	AbD Serotec
	CD16/32	Rat	1:1,000	553142	BD Pharmingen
	CD68	Rat	1:1,000	MCA1957	AbD Serotec
	Galectin-3	Rat	1:500	125402	BioLegend
	GFAP	Mouse	1:6,000	G3893	Sigma
	Iba1	Rabbit	1:500	GTX100042	GeneTex
	P2RY12	Rat	1:50	848001	BioLegend
	pHH3	Rabbit	1:500	06-570	EMD Millipore
	TREM2	Sheep	1:400	AF1729	R&D Systems
Secondary antibodies	Goat IgG	Alexa Fluor 568	1:1,000	A11057	Invitrogen
	Goat IgG	Alexa Fluor 488	1:1,000	A11055	Invitrogen
	Mouse IgG	Alexa Fluor 555	1:1,000	A31570	Invitrogen
	Rabbit IgG	Alexa Fluor 488	1:1,000	A21206	Invitrogen
	Rabbit IgG	Alexa Fluor 568	1:1,000	A10042	Invitrogen
	Rat IgG	Alexa Fluor 555	1:1,000	A21434	Invitrogen
	Rat IgG	Alexa Fluor 647	1:1,000	A21247	Invitrogen
	Sheep IgG	Alexa Fluor 488	1:1,000	A11015	Invitrogen
	Goat IgG	Biotinylated	1:500	BA-9500	Vector Laboratories
	Rabbit IgG	Biotinylated	1:500	BA-1000	Vector Laboratories
	Rat IgG	Biotinylated	1:500	BA-4001	Vector Laboratories
	Sheep IgG	Biotinylated	1:500	BA-6000	Vector Laboratories
HRP-conjugated Streptavidin			1:500	SA-5004	Vector Laboratories
DAPI			1:10,000	D9542	Sigma–Aldrich

### Double and Triple Immunohistochemistry

Double and triple immunolabeling combining (1) TREM2, ApoE or pHH3 with (2) either Iba1, CD68, CD16/32, Galectin-3, pHH3, P2RY12, GFAP or CD11b, and (3) GFAP were performed as follows: sections were washed and blocked for 1 h with blocking solution 3 (BS3) containing 0.2% gelatin in TBS with 0.1% Triton X-100 (TBS-T0.1%) or with BS2 for pHH3. Later, sections were incubated with sheep anti-TREM2 for 48 h at RT, or either goat anti-ApoE antibody or rabbit anti-pHH3 ON at 4°C plus 1 h at RT. After several washes with TBS-T0.1%, sections were incubated with the corresponding secondary antibody being anti-sheep Alexa-Fluor (AF) 488, anti-goat AF568, anti-goat AF488 in the case of the double immunostaining combining ApoE with GFAP, or anti-rabbit AF488 for 1 h at RT (Table 1). Afterward, sections were washed and incubated ON at 4°C plus 1 h at RT with rabbit anti-Iba1, rabbit anti-pHH3, rat anti-CD68, rat anti-CD16/32, rat anti-Galectin-3, rat anti-CD11b antibodies, or mouse anti-GFAP (Table 1). After several washes, sections in each combination were incubated for 1 h at RT with the corresponding secondary conjugated antibodies anti-rabbit AF568, anti-rat AF555 anti-rat AF647, or anti-mouse AF555 secondary antibodies (Table 1). For the triple IHC protocol, these last steps were repeated using a mouse anti-GFAP antibody and secondary anti-mouse AF488 conjugated antibody (Table 1). On the other hand, in the case of TREM2 and P2RY12 double immunohistochemistry, sheep anti-TREM2 and rat anti-P2RY12 primary antibodies were incubated simultaneously 48 h at RT (Table 1), and after washes, secondary anti-sheep AF488 antibody was firstly incubated for 1 h at RT, followed by a second incubation with an anti-rabbit AF568 antibody for 1 h at RT (Table 1). Finally, in double ApoE and Iba1 or GFAP immunostaining, sections were treated for a diminishing fluorescence background as described in Schnell et al. (1999). Thus, sections were dipped in distilled water, treated with CuSO_4_ (10 mM) in ammonium acetate buffer (50 mM CH_3_COONH_4_, pH 5.0) for 90 min, and rinsed again with distilled water before mounting.

Additionally, microglial proliferation was determined by BrdU staining. DNA was denatured by first incubating sections in 0.082 N HCl for 10 min at 4°C and then for 30 min in 0.82 N HCl at 37°C. Sections were subsequently rinsed with borate buffer (pH 8.5) and 0.5% Triton X-100 in TBS. Afterward, sections were incubated for 1 h at RT using BS2, and the anti-BrdU antibody was added and incubated ON at 4°C plus 1 h at RT (Table 1). After several washes with TBS-T1%, the corresponding conjugated anti-rat AF555 secondary antibody was added for 1 h at RT (Table 1). Then, the anti-Iba1 antibody was incubated ON at 4°C plus 1 h at RT, with a final 1 h incubation at RT of anti-rabbit AF488 conjugated antibody (Table 1). All double-labeled sections were nuclei stained with DAPI (Table 1) for 5 min, before being coverslipped with Fluorescence Mounting Medium (S-3023; Dako).

### Brightfield Microscopy Quantification

Quantitative analysis was performed on sections stained with single immunohistochemistry for TREM2, CD68, CD16/32, and pHH3. For each immunostaining, three to five WT, GFAP-IL6Tg, and GFAP-IL10Tg animals per time post-lesion and experimental lesion model were analyzed.

In the PPT model, images of two central hippocampal sections containing the deafferented molecular layer (ML) of the dentate gyrus (DG) for each lesioned animal, and the equivalent area for NL mice, were captured. A total of three photographs were obtained for each hippocampus, at 40× magnification in the case of TREM2 and 20× magnification for CD16/32 and CD68 analysis. For the FNA model, at least three representative sections from the brainstem of each animal, containing the central part of the contralateral and the ipsilateral FN, were photographed at 10× magnification. All images were obtained using a DXM 1200F Nikon digital camera joined to a brightfield Nikon Eclipse 80i microscope and the corresponding ACT-1 2.20 software (Nikon Corporation). In both models, quantification of each photograph was done by using the AnalySIS^®^ software, in which the threshold corresponding to the staining was manually set. In the case of TREM2, the threshold was set to quantify only TREM2 cellular staining, excluding the background staining corresponding to soluble TREM2 (sTREM2). Analysis of each photograph resulted in the percentage of area covered by the immunolabeling (% Area) and the intensity of the immunoreaction (Mean Gray Value Mean). Then, the AI index was calculated by multiplying the percentage of the immunolabeled area and the Mean Gray Value Mean (Acarin et al., 1997). Results of the AI index and intensity were expressed in arbitrary units.

In the case of PPT, quantification of microglial cell proliferation was carried out on hippocampal sections immunostained for the mitotic marker pHH3 in NL and lesioned animals at 2, 3, and 7 dpl. The number of pHH3+ microglial cells in the whole ML of the DG were manually counted using a 20× objective. Data were averaged and represented as pHH3+ cells/mm^2^.

For the FNA model, pHH3 quantification was done in NL and lesioned animals at 3, 7, 14, 21, and 28 dpl. The number of pHH3+ cells in the FN were manually counted using a 20× objective. Then, all FN counted were photographed at 10×, and the area of each FN was quantified using ImageJ software (Wayne Rasband, National Institute of Health, Bethesda, MD, USA). pHH3 cell density was calculated from the total pHH3+ cells/FN area for each animal, and results were expressed as pHH3+ cells/mm^2^.

### Confocal Microscopy Quantification

To evaluate the percentage of ramified microglial TREM2+ cells for the PPT model, sections immunostained with Iba1 and TREM2 were used. A minimum of three WT, GFAP-IL6Tg, and GFAP-IL10Tg animals euthanized at 3 dpl were quantified. In this case, the total ML of DG was photographed at 40× magnification with a Zeiss LSM 700 confocal microscope. From the total amount of microglial Iba1+ cells contained in the deafferented ML of the DG, ramified microglial TREM2+ cells were counted using the “Cell counter” plug-in from ImageJ software. Results were expressed as a percentage of Iba1+ cells containing TREM2 in their ramifications from the total number of Iba1+ cells (% TREM2 + Iba1+ cells/total Iba1+ cells).

In the FNA model, quantification of microglial clusters expressing TREM2 and microglial TREM2 ramified clusters was performed by analyzing the double immunofluorescence for Iba1 and TREM2. A minimum of three WT, GFAP-IL6Tg, and GFAP-IL10Tg animals at 14 and 21 dpl were used. The number of total microglial clusters in the FN, identified by Iba1+ staining, expressing TREM2 in microglial ramifications were manually counted using a 40× and 63× objective with a Zeiss LSM 700 confocal microscope. Results were expressed in percentage as TREM2 + Iba1+ microglial clusters from the total amount of Iba1+ clusters (% TREM2 + Iba1+ microglial clusters/total Iba1+ clusters).

### Tissue Processing for Protein Quantification

For quantifying sTREM2, NL, 3 dpl after PPT (*n* = 6–8) and 21 dpl axotomized WT animals (*n* = 10) were used. Animals were deeply anesthetized with a solution of ketamine (80 mg/kg) and xylazine (20 mg/kg) injected intraperitoneally at a dose of 0.015 ml/g and perfused intracardially for 1 min with cold 0.1 M DPBS (pH 7.4, 14190-094; Thermo Fisher Scientific, Waltham, MA, USA). Then, the brain was removed from the skull, and for NL and PPT-lesioned animals, the hippocampus, was dissected out. For FNA-lesioned animals, the brain was excised and two 0.5-mm-thick coronal slices were obtained from the brain trunk using a Mouse Brain Matrix (Zivic Instruments). For each slice, the dorsal inferior half was cut with sterile knives, and tissue containing the contralateral and ipsilateral FN was divided. Samples were immediately snap-frozen individually in liquid nitrogen, and stored at −80°C. The tissue of two hippocampi or three FN was pooled for protein extraction. Briefly, protein extraction was performed by using a mechanical homogenizer to disrupt tissue in lysis buffer, containing 25 mM HEPES, 2% Igepal, 5 mM MgCl_2_, 1.3 mM EDTA, 1 mM EGTA, 0.1 M PMSF, and protease (1:100, P8340; Sigma–Aldrich) and phosphatase inhibitor cocktails (1:100, P0044; Sigma–Aldrich), and allowing solubilization for 2 h at 4°C. Afterward, samples were centrifuged at 6,500 *g* for 10 min at 4°C in a microcentrifuge (5415R centrifuge, Eppendorf) and the supernatants were collected and frozen.

For separating the soluble protein fraction, each protein lysate was ultracentrifuged at 107,000 *g* for 30 min at 4°C in a Sorvall MTX 150 Series Micro-Ultracentrifuge. After ultracentrifugation, supernatants were collected and concentrated by using microcentrifuge cellulose filter units (MRCPRT010, Merck Millipore). Resulting protein lysates were collected and total protein concentration was determined with a commercial Pierce BCA Protein Assay kit (#23225; Thermo Fisher Scientific, Waltham, MA, USA) according to manufacturer’s protocol.

### Enzyme-Linked Adsorbent Immunoassay for sTREM2 Detection

Quantification of sTREM2 was performed by following the manufacturer’s instructions of a Mouse TREM2 ELISA kit (ABIN429539, Antibodies-online). Briefly, standards and the corresponding samples were incubated at 2.5 μg/μl in a pre-coated TREM2 ELISA plate. After reagent incubations, and the addition of TMB substrate and Stop solution, results were read at 450 nm in the microplate reader Varioskan^TM^ Lux (Thermo Fisher Scientific, Waltham, MA, USA).

### Isolation of Myelin and Fluorescent Labeling With pHrodo^TM^ Green STP Ester

Myelin was isolated following the protocol described by Rolfe et al. (2017). Briefly, six WT animals were dislocated under the effects of anesthesia (dose of 0.015 ml/g of 80 mg/kg ketamine and 20 mg/kg xylazine solution, injected intraperitoneally). Brains were removed quickly and cut into little pieces in a cold 0.32 M sucrose solution prepared in 0.1 M Tris.Cl buffer. After homogenization with a mechanical homogenizer, myelin was separated by using a sucrose gradient in an ultracentrifuge at 105,000 *g* for 45 min at 4°C (70Ti Rotor, Sorvall). Myelin was isolated from the interphase, and after a hypoosmotic shock using Tris.Cl buffer and 0.32 M sucrose, pelleted myelin was weighted and reconstituted in PBS at a 100 mg/ml (w/v) suspension.

For myelin fluorescent labeling, conjugation with pHrodo-STP Ester Green^®^ (P35369, Thermo Fisher Scientific, Waltham, MA, USA)—with excitation at 505 nm and emission at 525 nm—was used following the procedure described by Greenhalgh et al. (2018). Briefly, the pHrodo dye was dissolved in 75 μl dimethyl sulfoxide (DMSO, Sigma–Aldrich), and afterward, 18.5 μl of myelin in a concentration of 15 mg/ml was mixed with 25 μl of pHrodo and resuspended in 206.5 μl of PBS (pH 8.0). After 45 min of incubation at RT, with agitation and protected from light, labeled myelin was spun down, resuspended in PBS (pH 7.4), and stored at −80°C.

### Myelin Phagocytosis Assay and Flow Cytometry

The capacity of TREM2+ microglia in PPT-lesioned animals (at 3 dpl) and axotomized animals (21 dpl) to phagocyte myelin debris was analyzed by isolating cells with Percoll and performing a myelin phagocytosis assay followed by a flow cytometry procedure (Almolda et al., 2009; Greenhalgh et al., 2018). Briefly, anesthetized animals (dose of 0.015 ml/g of an 80 mg/kg ketamine and 20 mg/kg xylazine solution, injected intraperitoneally) were intracardially perfused for 1 min with 0.1 M DPBS. The brain was removed and the area of interest, either hippocampus or FN, was dissected out as described above. For each tube, an individual lesioned hippocampus, two NL hippocampus, or a pool of two either lesioned or contralateral NL FN were used. To obtain a cell suspension, samples were dissociated through 160 and 70 μm meshes and digested for 30 min at 37°C using type IV collagenase (17104-019; Life Technologies, Carlsbad, CA, USA) and DNAsa I (D5025; Sigma–Aldrich, St. Louis, MO, USA). Subsequently, each cellular suspension was centrifuged at RT for 20 min at 2,400 rpm in a discontinuous density Percoll gradient (17-0891-02; Amersham-Pharmacia, UK) between 1.03 and 1.08 g/ml using a Heareus Multifuge 3L-R centrifuge (ThermoFisher). Myelin in the upper layer was removed, and cells in the interphase and the clear upper-phase were collected and washed in PBS + 2% FBS. Afterward, the myelin phagocytosis assay was performed as follows: cells were resuspended in DMEM-F12 medium (ref. 31330-038; Thermo Fisher Scientific, Waltham, MA, USA) + 10% FBS, then 3 μg of pHrodo Green-labeled myelin were added, and samples were incubated at 37°C for 4 h. For negative controls, the same procedure was performed without adding myelin, or by resuspending cells in PBS + 2% FBS and preserving at 4°C. After myelin incubation, cells were washed one time with PBS + 2% FBS at 1,200 rpm for 10 min. Then, cells were blocked for Fc receptors by incubating them in a solution of purified CD16/32 diluted in PBS + 2% FBS, for 10 min at 4°C (Table 2). Afterward, cells were labeled for 30 min at 4°C with the combination of the following surface antibodies: anti-CD11b-APCCy7, anti-CD45-PerCPCy5, and anti-TREM2-PE (Table 2). In parallel, isotype-matched control antibodies for the different fluorochromes (BD Pharmingen, Switzerland) and samples that were not incubated with myelin were used as the negative control, while a cell suspension of splenocytes and cerebellum of GFAP-IL6Tg animals as the positive control. Finally, cells were acquired using a FACS Canto flow cytometer (Becton Dickinson, San Jose, CA, USA) in the corresponding fluorescent channels, and pHrodo-Green labeled myelin phagocytosed by microglia could be captured in the FITC channel. Results were analyzed using the FlowJo^TM^ software (FlowJo LLC).

**Table 2:** List of antibodies used in flow cytometry.

Target antigen		Format	Dilution	Catalog number	Manufacturer
Fc blocker	CD16/32	Purified	1:250	553142	BD Biosciences
Primary antibodies	CD11b	APC-Cy7	1:400	557657	BD Biosciences
	CD45	PerCP	1:400	557235	BD Biosciences
	TREM2	PE	1:400	FAB17291P	R&D Company

### Statistical Analysis

Statistics were performed using Graph Pad Prism^®^ software (Graph Pad Software Inc.) and results were expressed as Mean ± Standard error of the mean (SEM). Appropriate statistical tests were used for each condition: for time-course dynamics, one-way ANOVA with multiple comparison *post hoc* Tukey’s test was used, for microglial cell density Dunnet’s *post hoc* test was used instead, for sTREM2 comparison and flow cytometry analysis either unpaired or paired Student’s *t*-test was used, and finally, two-way ANOVA with multiple comparison *post hoc* Sidak’s test was used to compare Tg mouse lines with their corresponding WT.

## Results

### TREM2 Is Upregulated in the Denervated ML of the DG After PPT

**Figure 1:**
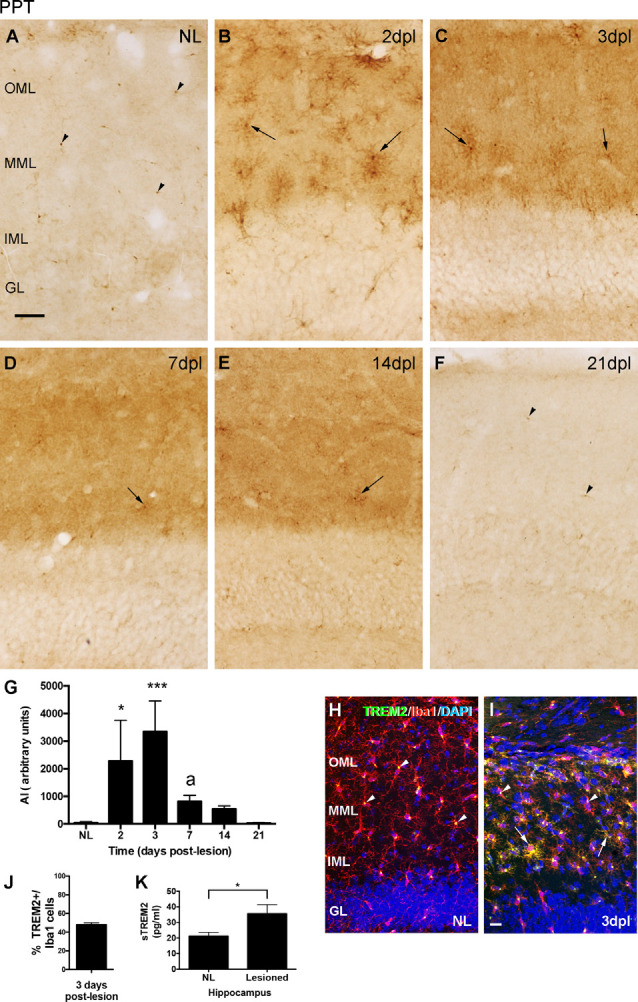
Temporal pattern of Triggering Receptor Expressed on Myeloid cells-2 (TREM2) changes in microglial cells after perforant pathway transection (PPT). **(A–F)** Representative images showing TREM2 staining in the granular (GL) as well as the inner, medial and outer molecular layers (IML, MML, and OML, respectively) of the dentate gyrus (DG) in non-lesioned (NL) **(A)** and PPT-lesioned mice from 2 to 21 dpl **(B–F)**. Note that, while in NL and at 21 dpl TREM2 was only depicted as small rounded morphologies (arrowheads), from 2 to 14 dpl ramified TREM2+ cells were also observed (arrows). **(G)** Graph showing the quantification of the AI index for TREM2 along the different time-points. Note the higher level of TREM2 at 2 and 3 dpl compared to NL and the posterior decrease from 3 to 7 dpl (one-way ANOVA, time effect *p* < 0.0001; *post hoc* Tukey’s test *p < 0.05, ***p < 0.001 respect to NL, a *p* < 0.01 respect to the previous time-point). **(H,I)** Double immunohistochemistry showing microglial cells labeled with Iba1 (red) and TREM2 (green). Note that in NL animals, TREM2 is confined to areas around the nucleus of some Iba1+ cells (arrowheads), whereas at 3 dpl, additionally, some prolongations of Iba1+ microglial cells (arrows) were also observed. **(J)** Graph showing the quantification of the percentage of TREM2+ cells, -those showing TREM2 staining in ramifications-, from total Iba1+ cells at 3 dpl. **(K)** Graph showing the quantification of sTREM2 (pg/ml) in the hippocampus of NL and PPT-lesioned mice at 3 dpl. A significant increase in the amount of sTREM2 is detected in the lesioned hippocampus (unpaired Student’s *t*-test, *p < 0.05). Scale bar **(A–F)** = 50 μm; **(H,I)** = 50 μm.

The immunohistochemical study of TREM2 expression after PPT showed that, in comparison to the low to undetectable TREM2 expression in the ML of NL animals, a significant upregulation in TREM2 immunolabeling in both the medial molecular layer (MML) and the outer molecular layer (OML) of the DG was observed (Figures 1A–F). A notable increase in TREM2 immunolabeling was found between 2 and 3 dpl, which sharply declined afterward, at 7 and 14 dpl. At 21 dpl, levels of TREM2 were comparable to basal conditions (Figure 1G).

Co-localization studies indicated that all TREM2+ cells corresponded to microglia/macrophages, as they co-labeled with Iba1 at all time-points analyzed (Figures 1H,I). Moreover, variations in the cellular location of TREM2 after injury were observed. Whereas in basal conditions TREM2 was found concentrated in the cytoplasm, in a position adjacent to the nucleus of microglial cells (arrowheads in Figure 1H); at 2 and 3 dpl, a subpopulation of microglial cells showed TREM2 expression in their ramifications (Figure 1I). Quantification of TREM2+/Iba1+ microglia cells at 3 dpl revealed that approximately half of microglia co-expressed TREM2 and Iba1 in their ramifications (48.1 ± 1.96%, in Figure 1J). From 3 dpl onwards, microglial cells expressing TREM2 in their ramifications decreased progressively. Interestingly, at 7 and 14 dpl, only a few scattered microglial processes were labeled, being TREM2 expression mostly restricted again into the region next to the nucleus. At 21 dpl, TREM2 was completely restricted in a position adjacent to the nucleus, like in basal conditions (Figure 1F). Notably, from 2 to 14 dpl, also a diffuse staining of sTREM2 covering the neuropil of both the MML and the OML was observed (Figures 1B–E). Quantification of sTREM2 at 3 dpl showed an increase of this protein fraction in the lesioned compared to the NL hippocampus (Figure 1K).

### TREM2 Is Upregulated In the Lesioned FN After FNA

**Figure 2:**
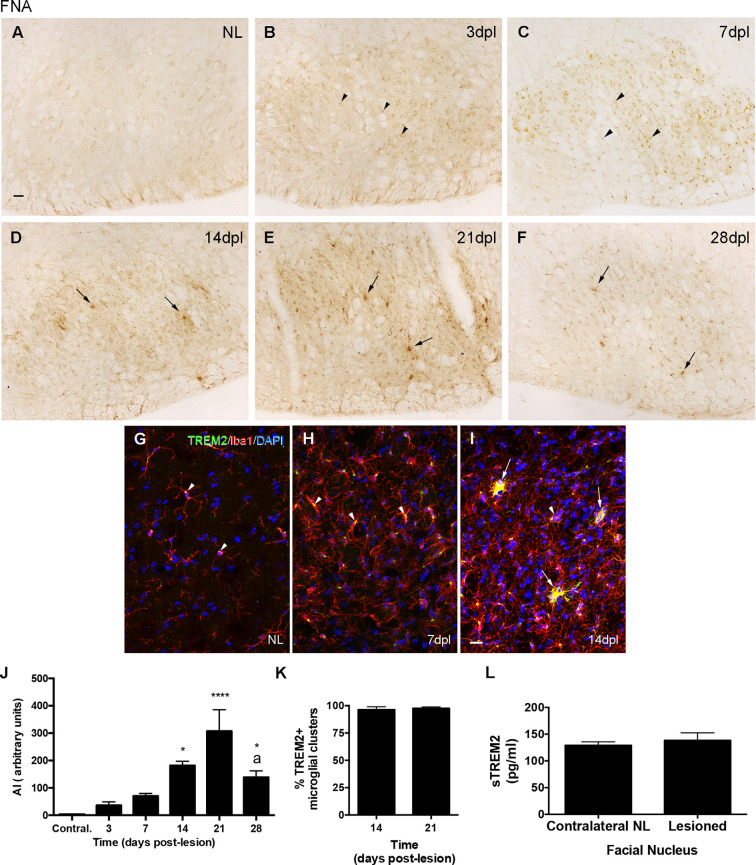
Temporal pattern of TREM2 changes in microglial cells after facial nerve axotomy (FNA). **(A–F)** Representative images showing TREM2 staining in the contralateral NL, as well as the ipsilateral sides of the FN from 3 to 28 dpl. From NL to 7 dpl TREM2 is mainly restricted to a perinuclear location (arrowheads), whereas from 14 dpl onwards TREM2 is extended to microglia ramifications and clusters (arrows). **(G–I)** Double immunohistochemistry showing microglial cells positive for TREM2 (green) and Iba1 (red). Note that in both the NL side and at 7 dpl, TREM2 is confined to areas around the nucleus of some Iba1+ cells (arrowheads), whereas at 14 dpl expression was detected in microglial prolongations in some microglial clusters (arrows). It is important to note that occasional Iba1 + TREM2-clusters were found at this time-point (arrowheads in **I**). **(J)** Graph showing the quantification of the AI index for TREM2 along the different time-points. Note the increase of TREM2 until 21 dpl and the later decrease (one-way ANOVA, time effect *p* < 0.0001; *post hoc* Tukey’s test *p < 0.05, ****p < 0.001 compared to NL; a *p* < 0.01 compared to the previous time-point). **(K)** Graph showing the percentage of microglial clusters containing TREM2+ microglial cells with expression in their ramifications at 14 and 21 dpl. **(L)** Graph showing the quantification of sTREM2 (pg/ml) in the FN of contralateral NL and the corresponding ipsilateral FN of mice lesioned at 21 dpl. No significant differences in the amount of sTREM2 are detected (paired Student’s *t*-test, *p* = 0.61). Scale bar **(A–F)** = 30 μm; **(G–I)** = 50 μm.

After FNA, the immunohistochemical study of TREM2 expression showed an upregulation of TREM2 in the ipsilateral FN along the different time points, in comparison to the NL contralateral side, where the levels of TREM2 were very low (Figures 2A–F). TREM2 immunolabeling increased from 3 dpl onwards, showed a maximum at 21 dpl, and decreased thereafter at 28 dpl, although levels were still higher than in the contralateral NL FN (Figure 2J).

Double-immunolabeled sections with TREM2 and Iba1 demonstrated that, in the NL contralateral side, TREM2 expression was concentrated in the cytoplasm of microglia, in a position adjacent to the nucleus (arrowheads in Figure 2G). In the ipsilateral side, at 3 and 7 dpl, TREM2 expression remained located like in basal conditions although levels of expression were higher (arrowheads in Figure 2H). In contrast, at 14 and 21 dpl, TREM2 staining was additionally found in the ramifications of a subpopulation of microglial cells, forming part of the so-called microglial clusters or microglial nodules (arrows in Figure 2I), which consist of accumulations of three to four microglial cells commonly described in the time-course of FNA injury (Moran and Graeber, 2004). At these time-points, almost all microglial clusters in the FN showed TREM2+ ramified microglia (Figure 2K and arrows in Figure 2I). In a similar way to the observations of PPT injury, low diffuse staining of sTREM2 covering the neuropil of the FN was observed, especially at 14 and 21 dpl. A comparison of the amount of sTREM2 fraction between NL and 21 dpl animals did not show a statistically significant increase in this protein fraction after lesion (Figure 2L).

### Phenotypic and Functional Characterization of TREM2+ Microglial Cells After PPT and FNA

TREM2 has been principally associated with microglial proliferation, phagocytosis, and DAM-triggering (Takahashi et al., 2005, 2007; Wang et al., 2015; Keren-Shaul et al., 2017; Krasemann et al., 2017). To determine if TREM2 microglial expression after PPT and FNA was related to these functions, we studied the dynamics of microglial proliferation (pHH3) and phagocytosis (CD16/32, CD68) in both models, and we compared it to the time course of TREM2 expression. We also performed co-localization studies with TREM2+ microglia using specific markers related to proliferation (pHH3, BrdU), phagocytosis (CD16/32, CD68, ApoE, Galectin-3), and DAM-triggering (Galectin-3, ApoE, and P2RY12), as well as a functional myelin phagocytosis assay. In this part of the study, we described as TREM2+ microglia the population appearing after both lesions that show an increase of TREM2 in microglial ramifications, according to our results.

#### Expression of Proliferation Markers

**Figure 3:**
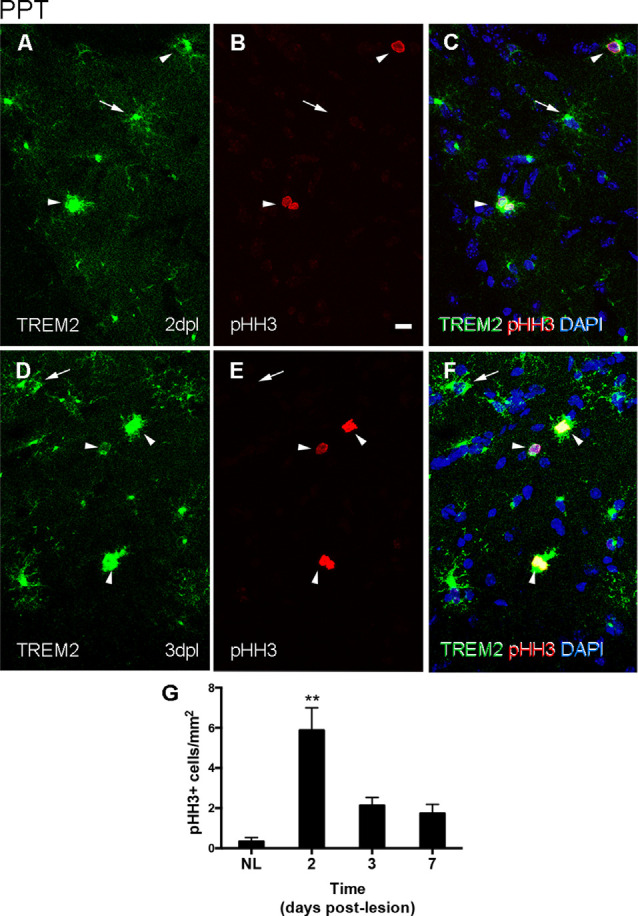
Determination of the proliferative activity of TREM2+ microglial cells after PPT. **(A–F)** Representative double-immunolabeled images combining TREM2 (green) and PHH3 (red) in the DG of PPT-lesioned animals at 2 dpl **(A–C)** and 3 dpl **(D–F)**. Note that at both time points PHH3+ cells showed membranal TREM2 expression in the ramifications and soma of microglial cells (arrowheads). However, not all TREM2+ microglial cells with expression in their ramifications were undergoing mitosis (arrows). **(G)** Graph of PHH3+ cell density (cell/mm^2^) in NL and PPT lesioned animals after 2, 3, and 7 dpl (one-way ANOVA, time effect *p* < 0.0001; *post hoc* Tukey’s compared to NL **p < 0.01). Scale bar **(A–F)** = 10 μm.

In the PPT paradigm, a subpopulation of TREM2+ cells in the MML and OML expressed pHH3, a mitotic marker, at both 2 and 3 dpl (Figures 3A–F), coinciding with the peak of microglial proliferation observed in this paradigm (Figure 3G). Despite all proliferative cells were TREM2+, the total amount of proliferating cells within the TREM2+ subpopulation was low.

**Figure 4:**
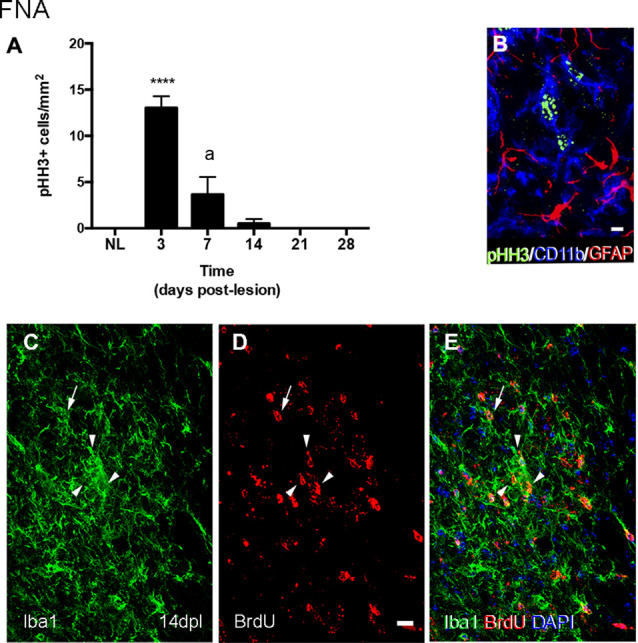
Determination of the proliferative activity in microglial clusters after FNA. **(A)** Graph showing the quantification of PHH3+ cell density in the FN after FNA. Note the massive increase of proliferative cells at 3 dpl, followed by a later decrease at 7 and 14 dpl, and the disappearance of proliferative cells from 21 dpl onwards (one-way ANOVA, time effect *p* < 0.0001; *post hoc* Tukey’s ****p < 0.001 compared to NL, a *p* < 0.001 compared to the previous time-point). **(B)** Representative triple immunofluorescence image combining PHH3 (green) with CD11b (blue) and GFAP (red). Note that all PHH3+ cells counted in the FN corresponded to microglia cells (CD11b+), but not astrocytes (GFAP). **(C–E)** Representative double-immunolabeled images combining TREM2 (green) and BrdU (red) in the FN of 14 dpl animals administered daily with BrdU. Proliferative BrdU+ microglial cells were observed inside microglial clusters (arrowheads) and outside them (arrows). Scale bar **(B)** = 5 μm; **(C–E)** = 10 μm.

After FNA, pHH3 cell counting showed a peak of proliferation at 3 dpl, when TREM2 was located intracytoplasmatically. The study of TREM2 with pHH3 showed that, at 14 and 21 dpl, a low number of proliferating pHH3+ microglial cells was detected (Figures 4A,B). Also, accumulative BrdU for 14 dpl indicated the presence of BrdU+ microglial cells in both the parenchyma and within clusters where, as previously described, TREM2+ cells were located. Proliferating cells showed variable BrdU intensity in the nucleus, indicating an uneven number of mitosis (Figures 4C–E).

#### Expression of the Phagocytic Markers CD16/32 and CD68

**Figure 5:**
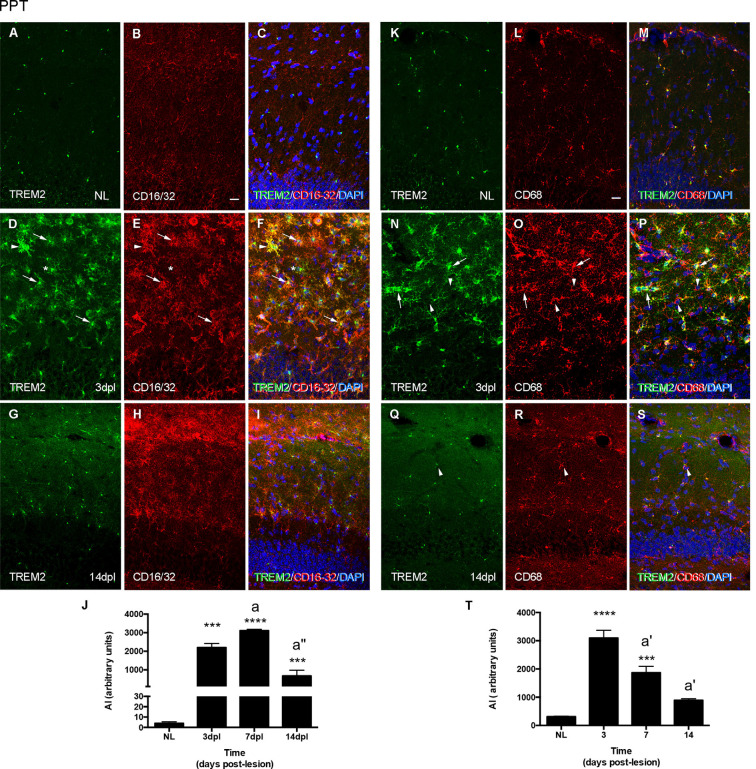
Analysis of CD16/32 and CD68 expression in TREM2+ microglial cells after PPT. **(A–I)** Representative double-immunolabeled images combining TREM2 (green) and CD16/32 (red) in the ML of the DG in NL **(A–C)** at 3 dpl **(D–F)** and 14 dpl **(G–I)**. Note that both TREM2 + CD16/32+ (arrows), some of them very intensely stained (arrowheads) and TREM2+ CD16/32- (asterisk) microglial cells were observed. **(J)** Graph showing the AI index of CD16/32 expression in NL and PPT-lesioned mice at 3, 7, and 14 dpl (One-way ANOVA, time effect *p* < 0.0001; *post hoc* Tukey’s test compared to NL ***p < 0.001, ****p < 0.0001; a *p* < 0.01, a^”^ *p* < 0.001 compared to previous time point). **(K–S)** Representative double-immunolabeled images combining TREM2 (green) and CD68 (red) in the DG of NL **(K–M)** and PPT-lesioned animals at 3 **(N–P)** and 14 dpl **(Q–S)**. Note that similarly to CD16/32, most TREM2+ ramified cells expressed also CD68 (arrows in **N–P**) at 3 dpl, whereas at 14 dpl TREM2 expression was restricted to the perinuclear area of CD68+ cells (arrowheads in **Q–S**). Also, some TREM2-CD68+ cells were found at 3 dpl (arrowheads in **N–P**). **(T)** Graph showing the AI index of CD68 expression in NL and PPT-lesioned mice at 3, 7, and 14 dpl (one-way ANOVA, time effect *p* < 0.0001; *post hoc* Tukey’s test compared to NL ***p < 0.001, ****p < 0.0001; a’ *p* < 0.01 compared to previous time point). Scale bar **(A–I,K–S)** = 50 μm.

The study of Fc-receptors CD16/32 and lysosomal CD68 expression in microglia during PPT and FNA time-courses provided a general assessment of changes in these phagocytic molecules. In the NL hippocampus, microglia express low levels of CD16/32, being not fully coincident with microglia expressing intracellular TREM2 (Figures 5A–C). After the PPT lesion, the majority of TREM2+ cells observed at 3 dpl, in the peak of microglia activation, presented variable CD16/32 staining (arrowheads and arrows in Figures 5D–F). At later time-points, 14 dpl, when TREM2 was restricted to the cytoplasm in a position adjacent to the nucleus, some microglia cells still maintained CD16/32 expression (Figures 5G–I). Furthermore, in NL hippocampus, CD68 and TREM2 were both located intracellularly and mostly coincident in microglia cells (Figures 5K–M). At 3 dpl, all TREM2+ microglia contained CD68, although CD68 expression was also found in other microglia, that is, CD68 is not exclusive to TREM2+ microglial subpopulation (arrowheads in Figures 5N–P). At 14 dpl, CD68 and TREM2 were again expressed intracellularly and mostly coincident in microglia (Figures 5Q–S).

In line with the above-mentioned observations, the dynamics of CD16/32 expression in the PPT lesion was sharply upregulated until 7 dpl and decreased thereafter at 14 dpl, although at this time point, levels of expression were still around 100-times higher than in NL animals (Figure 5J). In contrast, CD68 expression also was upregulated at 3 dpl but then progressively decreased from 7 to 14 dpl. At this later time-point, CD68 levels were still around two-times higher than NL mice (Figure 5T).

**Figure 6:**
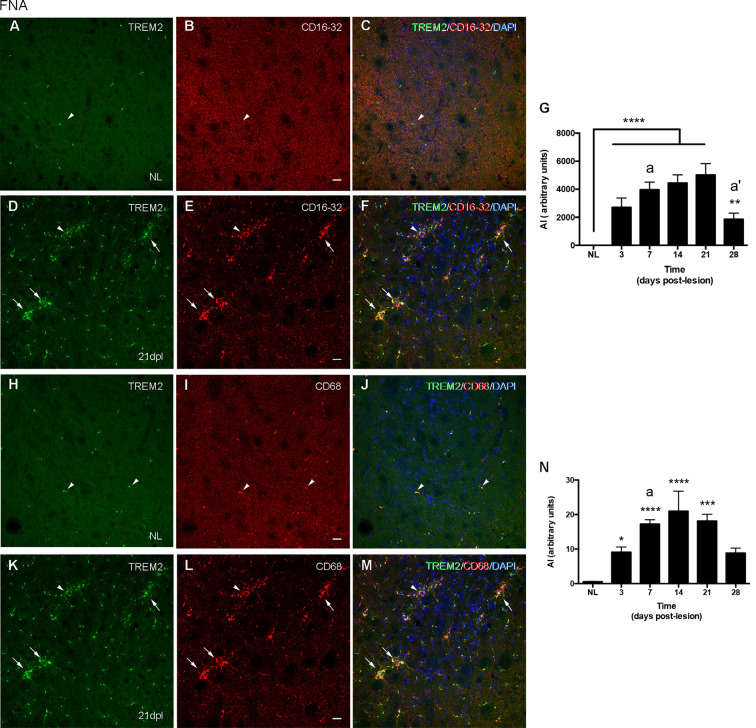
Analysis of CD16/32 and CD68 expression in TREM2+ microglial cells after FNA. **(A–F)** Representative double-immunolabeled images combining TREM2 (green) and CD16/32 (red) in the NL **(A–C)** and ipsilateral FN at 21 dpl **(D–F)**. Note that microglial clusters with low (arrowheads) or high (arrows) TREM2+ expression in their ramifications being CD16/32+ could be observed. **(G)** Graph showing the AI index of CD16/32 expression in the contralateral, NL, and the ipsilateral sides of the FN from 3 to 28 dpl (one-way ANOVA, time effect *p* < 0.0001; *post hoc* Tukey’s test compared to NL **p < 0.01, ****p < 0.0001, compared to the previous time-point a *p* < 0.05, a’ *p* < 0.0001). **(H–M)** Representative double-immunolabeled images combining TREM2 (green) and CD68 (red) in the NL **(H–J)** and ipsilateral FN of lesioned animals at 21 dpl **(K–M)**. Note that similarly to CD16/32, most CD68+ microglial clusters express high levels of TREM2 (arrows in **K–M**), but also some clusters with low levels of TREM2 were found to be CD68+ (arrowheads in **K–M**). **(N)** Graph showing the AI index of CD68 expression in NL and FNA-lesioned mice from 3 to 28 dpl (one-way ANOVA, time effect *p* < 0.01; *post hoc* Tukey’s test compared to NL *p < 0.05, ***p < 0.001, ****p < 0.0001, respect to the previous time-point a *p* < 0.05). Scale bar **(A–F,H–M)** = 50 μm.

In the contralateral NL FN, CD16/32 was not expressed in microglial cells (Figures 6A–C), while CD68 was located intracellularly and colocalized with intracellular TREM2 expression (Figures 6H–J). After FNA, the majority of clusters containing TREM2+ microglia also co-expressed both CD16/32 and CD68 (arrows in Figures 6D–F,K–M). However, the few microglial clusters that were TREM2- also contained microglial staining for CD16/32 and CD68 (arrowheads in Figures 6D–F,K–M).

When we studied the microglial expression pattern of CD16/32 and CD68 along the different time-points after FNA (Figures 6G,N), we found that both CD16/32 and CD68 levels increased rapidly at 3 and 7 dpl, respectively, remained elevated until 21 dpl, and decreased at 28 dpl (Figures 6G,N).

#### Expression of the Phagocytic Ligands ApoE and Galectin-3

According to results obtained in previous sections, we studied ApoE and Galectin-3 expression, both specific phagocytic ligands of TREM2 and DAM-associated molecules. The study was centered on the peak of TREM2 expression after PPT, around 3 dpl, and after FNA, at 14 and 21 dpl, compared to basal conditions.

**Figure 7:**
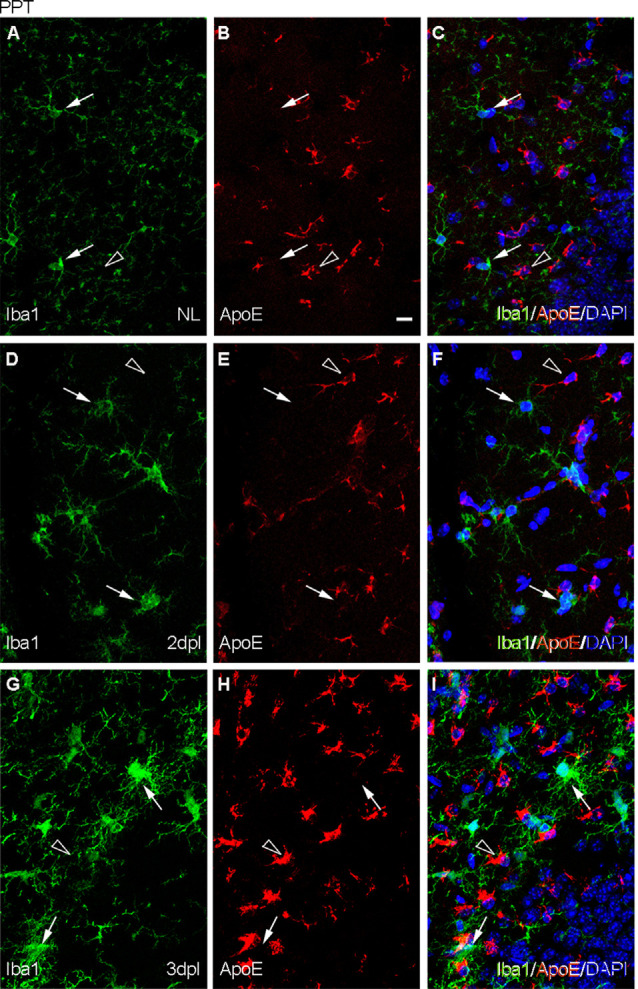
Analysis of the expression of TREM2 ligand ApoE after PPT. **(A–I)** Representative images of double immunofluorescence combining ApoE (red) and Iba1 (green) in the NL DG **(A–C)** and at 2 dpI **(D–F)** and 3 dpl **(G–I)** after PPT. Note that ApoE expression could be observed on astrocyte-like cells (empty arrowheads), but not in microglia (white arrows) in all the time-points. Scale bar **(A–I)** = 10 μm.

**Figure 8:**
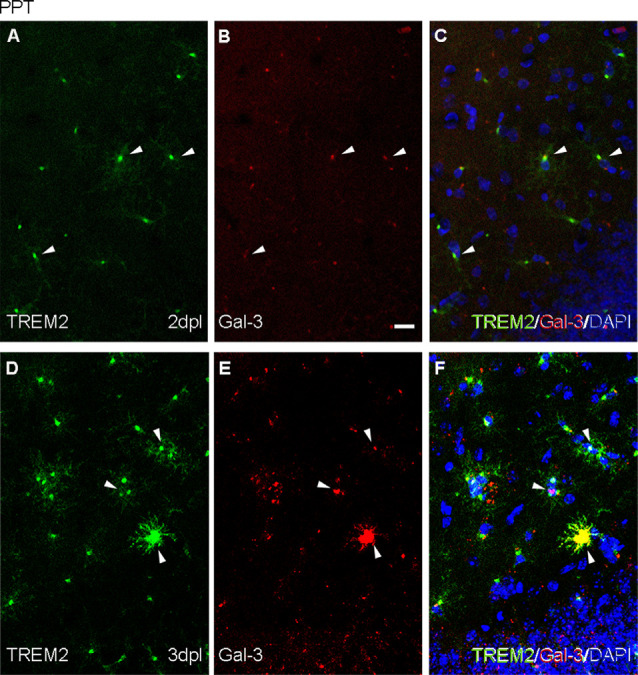
Analysis of the expression of TREM2 ligand Galectin-3 after PPT. **(A–F)** Representative double immunofluorescence images combining Galectin-3 (red) and TREM2 (green) at 2 dpI **(A–C)** and 3 dpI **(D–F)** after PPT. Occasional microglial cells showing TREM2 expression in their ramifications contained Galectin-3 (white arrowheads) as perinuclear staining in both time-points. Note that at 3 dpI some microglial cells also presented Galectin-3 in their ramifications, indicating an increase of Galectin-3 compared to 2 dpI. Scale bar **(A–F)** = 10 μm.

In the NL hippocampus, no expression of ApoE was observed in microglia, like similarly observed in the PPT paradigm at any time-point (Figures 7A–I), as its expression was in all cases restricted to astrocytes (Supplementary Figure 1). Moreover, *de novo* expression of Galectin-3 in some TREM2+ microglial cells was reported at 2 and 3 dpl (Figures 8A–F).

**Figure 9:**
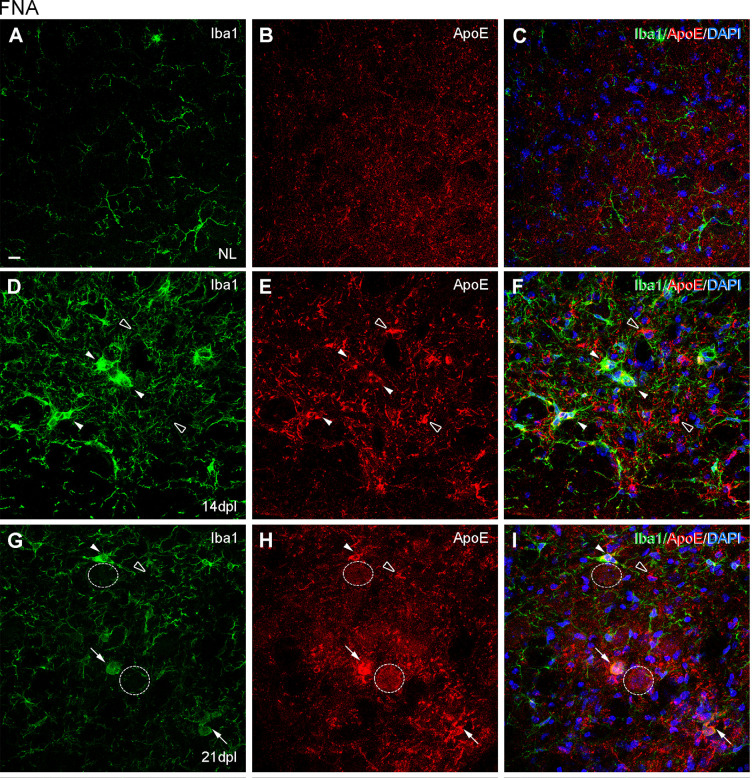
Analysis of ApoE expression in microglial clusters after FNA. **(A–I)** Representative double-immunolabeled images combining Iba1 (green) and ApoE (red) in the NL FN **(A–C)** and at 14 dpI **(D–F)** and 21 dpl **(G–I)**. In the lesioned FN, astrocyte-like cells were easily identified by strong ApoE staining (empty arrowheads). Note that at 14 and 21 dpl, ApoE was also present in microglial clusters (white arrowheads), and in some of them with high levels of ApoE staining (white arrows), although not all clusters were positive for ApoE (data not shown). Additionally, at 21 dpl, some FMN showed ApoE staining on their surface (dashed line circles). Scale bar **(A–I)** = 10 μm.

**Figure 10:**
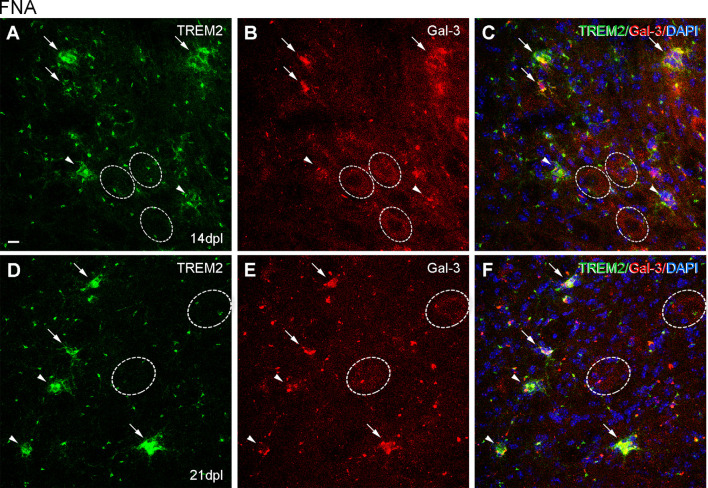
Analysis of Galectin-3 expression in microglial clusters after FNA. **(A–F)** Representative double-immunolabeled images combining TREM2 (green) and Galectin-3 (red) in the ipsilateral FN at 14 dpI **(A–C)** and 21 dpI **(D–F)**. Note that at 14 and 21 dpI, clusters displaying high intensity for Galectin-3 (white arrows) could show either high or low intensity for TREM2. Likewise, clusters presenting low Galectin-3 (white arrowheads) presented variable TREM2 intensity as well. No differences in microglial cluster Galectin-3 staining were seen between 14 and 21 dpl. FMN were also positive for Galectin-3 at 14 and 21 dpI, being more easily detectable at 14 dpI (dashed line circles). Scale bar **(A–F)** = 10 μm.

After FNA, the expression of ApoE was restricted to astrocytes in the contralateral NL side (Figures 9A–C; Supplementary Figure 2), but on the ipsilateral side, ApoE staining was observed in cells of TREM2+ microglial clusters at both 14 and 21 dpl (Figures 9D–I). Moreover, ApoE+ facial motor neurons (FMN, dashed line circles in Figures 9D–I) were observed. As far as Galectin-3 is concerned, *de novo* expression of this marker was found in some TREM2+ microglial clusters, although no clear relationship between Galectin-3 and TREM2 intensities was observed, since clusters with high TREM2 levels presented variable Galectin-3 intensity staining (arrows and arrowheads, Figures 10A–F). Additionally, Galectin-3 staining on the surface of isolated FMN was found at 14 and 21 dpl (dashed line circles in Figures 10A–F).

#### Expression of the Homeostatic Microglial Marker P2RY12

**Figure 11:**
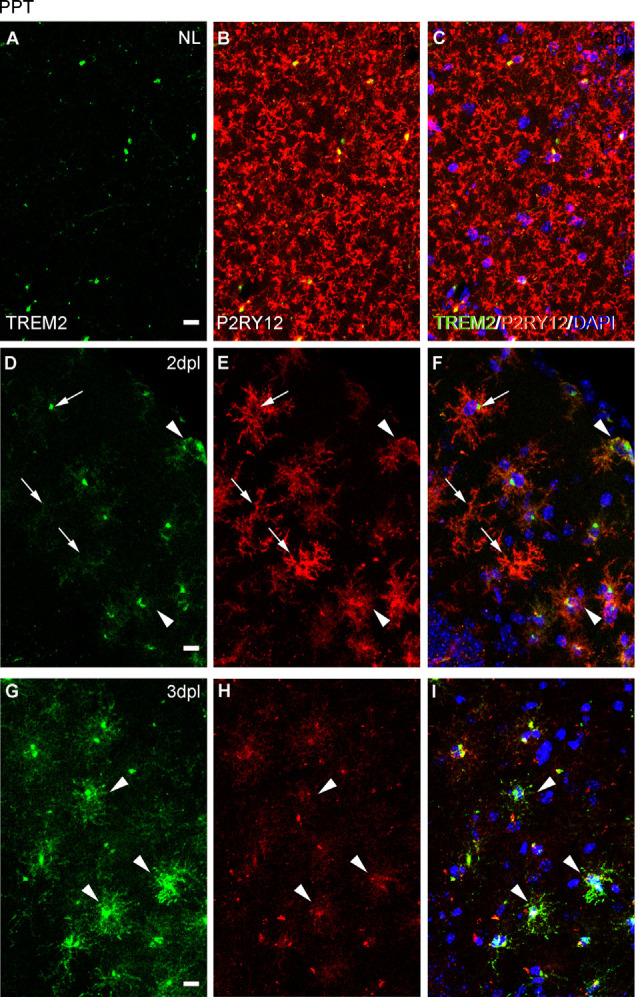
Analysis of P2RY12 expression in microglial cells after PPT. **(A–I)** Representative images showing P2RY12 expression in the DG of the hippocampus in NL conditions **(A–C)**, at 2 dpl **(D–F)**, and 3 dpl **(G–I)** after PPT. In NL conditions, TREM2 (in green) is expressed accumulated adjacent to the nucleus, and high levels of P2RY12 (in red) are found in all microglial cells. After lesion, at 2 dpl, most microglia show low levels of TREM2 in ramifications and high levels of P2RY12 expression in all microglial cells (arrows). Also, some microglia with high levels of TREM2 expression and low levels of P2RY12 microglia are observed (arrowheads), being the latter the microglial phenotype mostly seen at 3 dpl. Scale bar **(A–I)** = 10μm.

**Figure 12:**
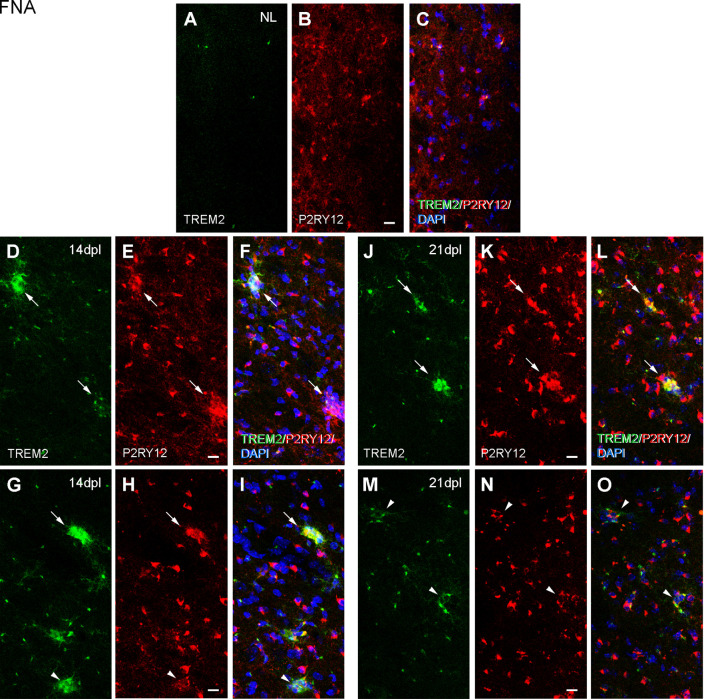
Analysis of P2RY12 expression in microglial clusters after FNA. **(A–O)** Representative double-immunolabeled images combining TREM2 (green) and P2RY12 (red) in the ipsilateral NL FN **(A–C)** and at 14 **(D–I)** and 21 dpI **(J–O)**. Note that clusters with high levels of P2RY12 (arrows) can present both high and low levels of TREM2. In the same way, clusters with low levels of P2RY12 (arrowheads) show high and low levels of TREM2. No differences were detected between all the time-points in terms of P2RY12 staining. Scale bar **(A–O)** = 10 μm.

Because ApoE and Galectin-3 molecules were upregulated, as it is seen in DAM, we were interested to observe whether the homeostatic receptor P2RY12 was downregulated, to further confirm similarities with the DAM phenotype. After PPT, in contrast to the high P2RY12 expression observed in all microglia of the NL DG; a gradual downregulation of P2RY12 levels was observed in TREM2+ microglial cells of the OML and MML until 3 dpl (arrowheads, Figure 11). However, in the FNA model, different levels of P2RY12 were reported in TREM2+ microglial clusters at both 14 and 21 dpl. Indeed, TREM2+ clusters showing either low or high P2RY12 were observed. Few TREM2- clusters located within the same region showed high P2RY12 levels (Figures 12A–O).

#### Microglial Phagocytosis of Myelin by TREM2+ Microglia After PPT and FNA

**Figure 13:**
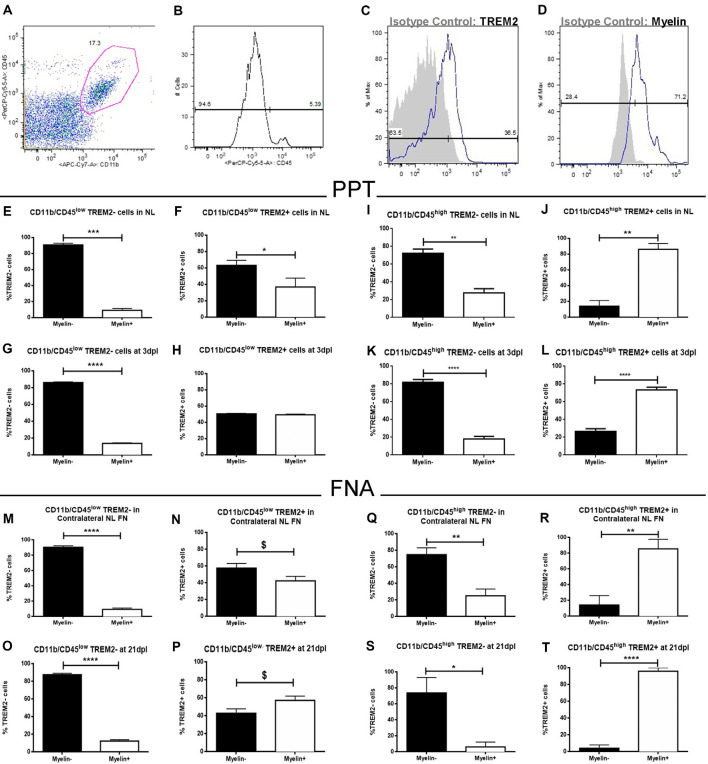
Flow cytometry analysis of myelin phagocytosis by TREM2+ microglia and macrophages from NL and lesioned hippocampus and FN after PPT and FNA. **(A)** Representative dot-plot of CD11b/CD45 expression in cells obtained from the hippocampus of NL animals. The selected region was the population of cells used in this study. **(B)** Representative histogram where populations of CD11b+/CD45^low^ cells (microglia) and CD11b+/CD45^high^ cells (macrophages) were defined. **(C,D)** Representative histogram-plot showing the expression of TREM2 **(C)** and Myelin **(D)** in the population of microglia/macrophages in comparison to the corresponding isotype control. **(E–H)** Graphs showing the percentage of TREM2− and TREM2+ cells phagocyting myelin in NL **(E,F)** and at 3 dpl **(G,H)** in CD11b+/CD45^low^ population after PPT. **(I–L)** Graphs showing the percentage of TREM2− and TREM2+ cells phagocyting myelin in NL **(I,J)** and at 3 dpl **(K,L)** in CD11b+/CD45^high^ population after PPT. **(M–P)** Graphs showing the percentage of TREM2− and TREM2+ cells phagocyting myelin in contralateral NL FN **(M,N)** and at 21 dpl **(O,P)** in CD11b+/CD45^low^ population after FNA. **(Q–T)** Graphs showing the percentage of TREM2− and TREM2+ cells phagocyting myelin in contralateral NL FN **(Q,R)** and at 21 dpl **(S,T)** in CD11b+/CD45^high^ population after FNA. **(E–T)** Results were represented as ± SEM (Unpaired Student’s *t*-test, ****p ≤ 0.0001, ***p ≤ 0.001, **p ≤ 0.01, *p ≤ 0.05, ^$^*p* ≤ 0.08).

To study the functional phagocytosis capacity of both TREM2+ and TREM2- microglia after PPT and FNA, we performed a myelin phagocytosis assay and flow cytometry analysis of CD11b+/CD45^low^ and CD11b+/CD45^high^ populations (Figures 13A–D). Our results show that in the CD11b+CD45^low^ population of NL hippocampus and FN, formed by microglia, TREM2- cells are phagocytosing myelin debris in low percentages, in all cases below 20% (Figures 13E,M). Interestingly, similar percentages are maintained after lesion (Figures 13G,O), despite the number of cells is increased in these conditions. Therefore, our results indicate that phagocytosis is not exclusive of TREM2+ cells within CD11b+/CD45^low^ cells but is a minor function within this population. On the contrary, when we studied CD11b+/CD45^low^/TREM2+ cells in NL hippocampus and FN, we could observe that phagocytosis by microglial cells is increased, and differences between Myelin- and Myelin+ cells observed within the TREM2- population are hardly maintained (Figures 13F,N). Furthermore, in the lesioned hippocampus, 50% of cells are phagocyting myelin (Figure 13H), whereas after FNA there is a tendency to show a higher percentage of phagocytic TREM2+ cells (Figure 13P). In the case of CD11b+/CD45^high^ populations, formed by macrophages or highly activated microglia, TREM2- cells show a similar phagocytic capacity than the one observed in CD11b+/CD45^low^ cells: high percentages of non-phagocytic cells are found in NL and lesioned hippocampus and FN (Figures 13I,K,Q,S). Observation of phagocytosis in CD11b+/CD45^high^/TREM2+ population showed a completely opposed behavior, as NL and lesioned hippocampus and FN contained elevated percentages, over 70%, of phagocytic cells (Figures 13J,L,R,T). Altogether, our results indicate that TREM2 is a receptor preferentially found on phagocytotic cells but it is not exclusive of them, as also TREM2- cells can phagocytose myelin.

### Transgenic Overproduction of Either IL-6 or IL-10 Modifies TREM2 Expression, Especially After Anterograde Degeneration (PPT Model)

Previous studies in our laboratory (Almolda et al., 2014; Villacampa et al., 2015; Recasens et al., 2019) using two Tg animal lines with an astrocyte-targeted expression of the pro-inflammatory cytokine IL-6 (GFAP-IL6Tg) and the anti-inflammatory cytokine IL-10 (GFAP-IL10Tg), demonstrated significant differences in the microglial activation pattern after PPT and FNA. These differences correlated with important modifications in lesion outcomes, either neuronal survival or axonal sprouting. Considering that the effect of cytokines into TREM2 expression is not known and that neuronal survival and axonal sprouting are conditioned to the microglial phagocytic activity which, according to our results, can be modified by TREM2 expression, we found of great interest to assess the regulation of cellular TREM2 expression in these Tg animals after both experimental models.

**Figure 14:**
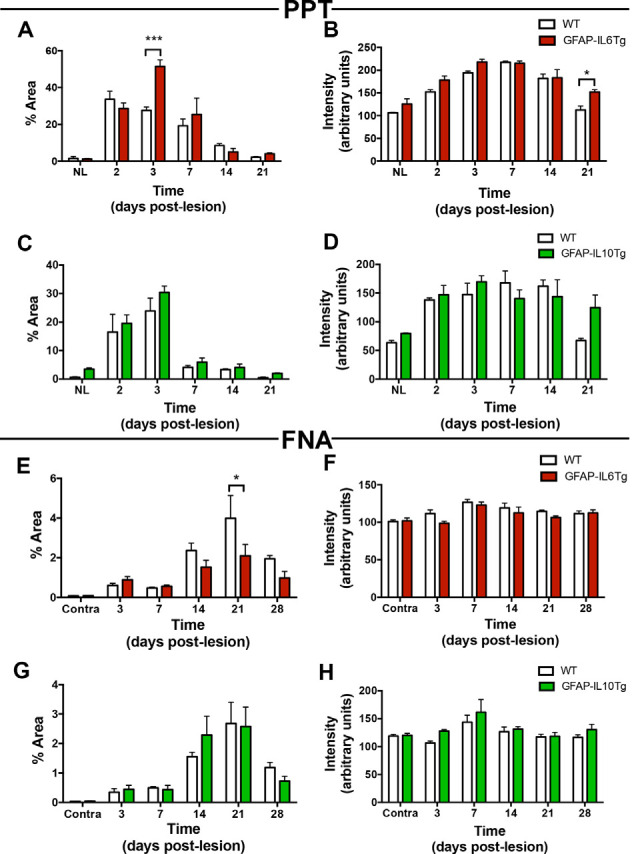
Temporal pattern of TREM2 expression in microglial cells in mice with astrocyte-targeted overexpression of IL-6 (GFAP-IL6Tg) or IL-10 (GFAP-IL10Tg) compared to their corresponding wild-type (WT) after PPT and FNA. **(A–D)** Graphs showing either the percentage of Area **(A,C)** or intensity **(B,D)** of TREM2 expression in the OML and MML of the DG of PPT-lesioned GFAP-IL6Tg, GFAP-IL10Tg, and their corresponding WT littermates. Statistically significant differences are observed between GFAP-IL6Tg and WT animals in the percentage of Area of TREM2 expression at 3 dpl (two-way ANOVA, time effect *p* < 0.0001, genotype *p* = 0.07; interaction time-genotype *p* < 0.01; Sidak’s multiple comparison test, ***p < 0.001) as well as the intensity of TREM2 expression (two-way ANOVA, time effect *p* < 0.0001, genotype *p* < 0.01; Sidak’s multiple comparison test, *p < 0.05). Also, differences between GFAP-IL10Tg and WT mice are shown in the percentage of Area of TREM2 expression (two-way ANOVA, time effect *p* < 0.0001, genotype *p* = 0.08; no significant differences were found with Sidak’s multiple comparison test) and the intensity of TREM2 expression (two-way ANOVA, time effect *p* < 0.0001, genotype *p* = 0.308; no significant differences were found with Sidak’s multiple comparison test). **(E–H)** Graphs showing either the percentage of Area **(E,G)** or intensity **(F,H)** of TREM2 expression in the ipsilateral FN of GFAP-IL6Tg and GFAP-IL10Tg lesioned animals, and their corresponding WT littermates. Differences in the percentage of Area (two-way ANOVA, time effect *p* < 0.0001, genotype effect *p* < 0.05; Sidak’s multiple comparison test, *p < 0.01) and intensity (two-way ANOVA, time effect *p* < 0.0001, genotype effect *p* = 0.06; no significant differences detected with Sidak’s multiple comparison test) of TREM2 in GFAP-IL6Tg compared to WT were found. In the case of GFAP-IL10Tg, no statistically significant differences between time-points were observed at neither the percentage of Area (two-way ANOVA, time effect *p* < 0.0001, genotype effect *p* = 0.883, no significant differences detected with Sidak’s multiple comparison test) nor the intensity (two-way ANOVA, time effect *p* < 0.01, genotype effect *p* = 0.06; no significant differences detected with Sidak’s multiple comparison test).

In NL conditions, we detected a qualitative increase of TREM2 intensity levels in the OML and MML of the hippocampus of both GFAP-IL6Tg and GFAP-IL10Tg mice; but no modifications were observed in the NL FN (Figures 14B,D,F,H; Supplementary Figures 3A,E,I,L).

**Figure 15:**
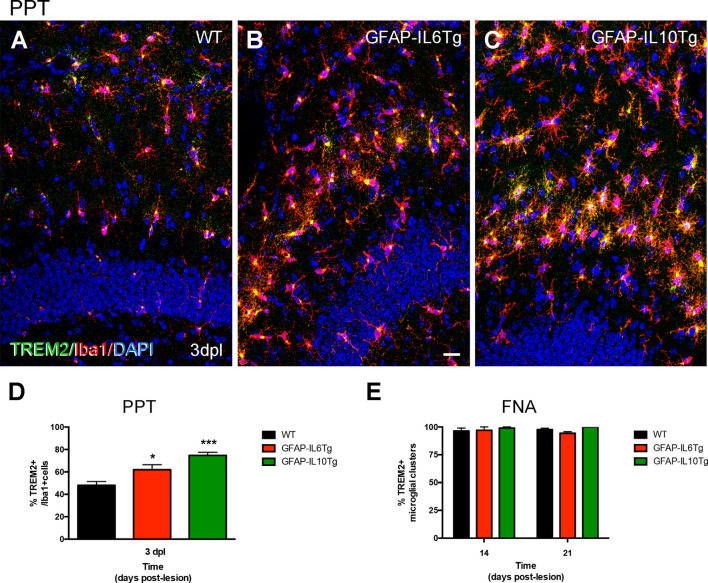
Quantification of TREM2+ microglia subpopulation after PPT and TREM2+ microglial clusters after FNA in GFAP-IL6Tg, GFAP-IL10Tg, and WT mice. **(A–C)** Representative double-immunolabeled images combining TREM2 (green) and Iba1 (red) in the ML of the DG at 3 dpl in WT **(A)**, GFAP-IL6Tg **(B)**, and GFAP-IL10Tg **(C)**. **(D)** Graph showing the percentage of TREM2+ ramified cells concerning the total number of Iba1 cells at 3 dpl in WT (48.1 ± 1.96%), GFAP-IL6Tg (61.97 ± 2.58%), and GFAP-IL10Tg (74.7 ± 1.91%). A higher percentage of TREM2/Iba1 cells are found in Tg mice concerning WT at 3 dpl (one-way ANOVA, *p < 0.01; *post hoc* Dunnet’s test, GFAP-IL6Tg vs. WT *p = 0.01, and GFAP-IL10Tg vs. WT ***p = 0.001). **(E)** Graph showing the percentage of microglial clusters with TREM2+ expression in their ramifications at 14 and 21 dpl after FNA in WT, GFAP-IL6Tg, and GFAP-IL10Tg. Scale bar **(A–C)** = 50 μm.

After PPT, a higher percentage of area occupied by TREM2 in the deafferented area of the ML was detected at 3 dpl in both Tg mice (Figures 14A,C; Supplementary Figures 3B,F). These differences were more pronounced in the GFAP-IL6Tg mice than in GFAP-IL10Tg mice compared to their respective WT. In line with these observations, at 3 dpl, a higher percentage of ramified TREM2+/Iba1+ microglial cells was found in GFAP-IL10Tg (74.7 ± 1.91%) and in GFAP-IL6Tg (61.97 ± 2.58%) concerning WT animals (48.1 ± 1.96%; Figures 15A–D).

After FNA, a significantly lower percentage of TREM2 stained area was observed at 21 dpl in GFAP-IL6Tg mice compared to WT mice (Figure 14E; Supplementary Figure 3K). Similar to WT mice, in GFAP-IL6Tg and GFAP-IL10Tg mice, all or almost all microglial clusters showed TREM2 staining in microglial ramifications at 14 dpl and at 21 dpl (Figure 15E).

## Discussion

In this work, we studied TREM2 regulation in microglia after axonal injury, in both anterograde and retrograde neurodegeneration models: the PPT and the FNA respectively. Results obtained demonstrate an upregulation of TREM2 expression in both models of axonal injury but following different patterns. In the PPT, TREM2+ cells appeared at early time-points (2 and 3 dpl), whereas after FNA TREM2+ microglia were observed mainly forming clusters at 14 and 21 dpl. Also, we identified singular subpopulations of TREM2+ microglia expressing CD16/32, CD68 as well as showing *de novo* expression of ApoE or Galectin-3 and diverse levels of the homeostatic P2RY12 molecule. Finally, using Tg animals, we demonstrated that the cytokine microenvironment, which modifies microglial activation, influenced TREM2 expression, especially in the PPT paradigm and under IL-6 overexpression. In the following paragraphs, we will discuss our findings with the current literature.

### TREM2 Is Increased in Activated Microglial Cells After Both PPT and FNA

TREM2 is a receptor found in the membrane of myeloid immune cells such as macrophages, osteoclasts, and microglia (Schmid et al., 2002; Colonna, 2003). Under basal conditions, TREM2 has been reported in microglial cells, both *in vitro* and *in vivo*, during development and in adulthood (Schmid et al., 2002; Chertoff et al., 2013). In agreement, we observed constitutive TREM2 expression in microglia in the FN and hippocampus of NL animals. In these regions most TREM2 labeling was restricted to a concentrated area in the cytoplasm, in a position adjacent to the nucleus of the microglial cells, coinciding with a previous study where the intracellular distribution was shown in two pools: a deposit in the Golgi complex and a population of exocytic vesicles, that would facilitate a continuous translocation to and recycled from the cell surface (Prada et al., 2006). This storage of intracellular TREM2 favors the continuous functional adaptation of these cells to the microenvironment and their rapid response to microglial activation. However, the fact that TREM2 shall be exposed to the membrane to interact with their ligands, lead us to study the TREM2+ microglial population appearing after lesions, showing elevated levels of TREM2 expression in ramifications, and according to our flow cytometry study, at least with partial TREM2 expression at the level of the cytoplasmic membrane.

In our study, upregulation of TREM2 expression could be observed in both models of axonal injury, being coincident with the previous general *in vivo* data in chronic (Jay et al., 2017a; Sayed et al., 2018) and acute CNS inflammatory injuries (Saber et al., 2017; Scott-Hewitt et al., 2017; Wu et al., 2017), as well as in other axonal injury models (Kobayashi et al., 2016; Tay et al., 2018). Additionally, an increase in sTREM2 could be found at the peak of TREM2 microglial expression after PPT, which was not detected after FNA. sTREM2 is the result of proteolytic TREM2 cleavage by ADAM10 and ADAM17 at H157–S158 peptide bond, resulting in its liberation. Despite little is known about the role of sTREM2, this fraction has been related to the potentiation of several microglial functions including pro-inflammatory cytokine production, migration, survival, and phagocytosis (Zhong et al., 2017, 2019).

Although both injury models presented expression of TREM2 in microglia, differences in the pattern of expression of this molecule were found between anterograde and retrograde degeneration models. Concretely, after PPT, TREM2 expression followed a pattern coincident with microglia activation (Recasens et al., 2019), but after FNA, TREM2 levels increased at 14–21 dpl and were restricted to microglial clusters that do not coincide with microglial activation, which starts at 3 dpl, peaks from 7 to 14 dpl, and is downregulated afterward (Villacampa et al., 2015). These results suggest that not all activated microglia express TREM2 and pointed towards a specific role of this receptor in microglial function. Differences in TREM2 expression could be related to the nature of the lesion (anterograde vs. retrograde) and microglial cell function along the time-course of both models. On one hand, PPT is a model that involves the complete transection of the perforant pathway, that produces an anterograde axonal and terminal degeneration of these axons in the outer 2/3 of the ML in the hippocampal DG. Due to this lesion, a rapid and robust microglial activation is induced in the DG at 2–5 dpl (Lynch et al., 1972; Jensen et al., 1999; Ladeby et al., 2005), whose principal function is to phagocyte myelin and neuronal debris (Nielsen et al., 2009), contributing to the structural reorganization and axonal collateral sprouting initiated around 7 dpl and being complete at 14 dpl (Deller et al., 2007). On the other hand, the FNA model involves a mechanical transection of the facial nerve, that leads to retrograde neuronal degeneration accompanied by a dramatic microglial activation. During early microglia activation and proliferation, from 3 to 7 dpl, TREM2 expression is mainly intracytoplasmatic and does not increase its expression in ramifications. Importantly, we observe an increase in intracellular TREM2 in this period, which could suggest a role for the intracellular TREM2 pool in microglia. Later, TREM2 is upregulated in specific structures called microglial clusters, first appearing at 14 dpl, located around FMN and involved in phagocytosis (Moran and Graeber, 2004).

The fact that TREM2 increases its expression in microglia coinciding in both models with the specific time-point related to phagocytosis, together with the expression of different markers, CD16/32, and CD68, involved in this function, points out to an especially relevant role of TREM2+ microglia after axonal injury in this function.

### TREM2+ Cells Expressed Markers Related to Proliferation and Phagocytosis After FNA and PPT

The phenotypic characterization of TREM2+ microglial cells showed expression of both proliferation and phagocytic markers after PPT, but only phagocytic markers after FNA.

In terms of proliferation, our results showed that, after PPT, all proliferating microglia were TREM2+. In previous reports in DAP12KO mice, a reduced microglial increase after spinal nerve transection (Kobayashi et al., 2016) was found. Also, TREM2KO mice showed reduced microglia proliferation during homeostasis and after injury (Cantoni et al., 2015; Poliani et al., 2015; Zheng et al., 2017), and impaired proliferation was observed in AD mouse models without TREM2 or harboring dysfunctional TREM2 variants (Jay et al., 2017a; Cheng-Hathaway et al., 2018; Meilandt et al., 2020). On the other hand, some authors demonstrated that lack of TREM2 did not affect microglia proliferation in microglial cell cultures (Noto et al., 2010) or after spinal nerve transection in DAP12KO (Guan et al., 2016). From our point of view, our current study demonstrates that the increase in TREM2+ microglia depends on the specific features of microglial response to axonal injury. In PPT, proliferation is coincident with microglia activation, and the lesion is rapidly rectified; while after FNA, activation and proliferation are immediately triggered, and phagocytosis of late degenerating FMN and injury resolution are delayed. Our BrdU study demonstrated that cells that were previously proliferating after FNA, and were not showing TREM2 expression at that time, were forming phagocytic microglial clusters, which are mainly TREM2+. Yet, further studies are needed to demonstrate a clear relationship between TREM2 expression and proliferation in axonal lesions.

Despite TREM2 phagocytic role has been extensively studied in acute and chronic neurodegenerative diseases models as AD or multiple sclerosis (reviewed in Deming et al., 2018; Karanfilian et al., 2020); to the best of our knowledge, this is the first study showing a link of TREM2 with phagocytic microglia after direct or primary acute axonal injury. As far as the possible role of TREM2+ microglial cells in phagocytosis is concerned, our results indicate an important implication of TREM2 in phagocytosis in both models, thus coinciding with the view of TREM2 as a receptor involved in the triggering of an “eat-me” signal (Neumann et al., 2009). After both PPT and FNA, most TREM2+ cells also expressed the phagocytic-associated molecules CD16/32 and CD68, and the myelin phagocytosis assay in TREM2+ microglia showed higher percentages of phagocytosis of myelin debris compared to TREM2- cells. In our results, the pattern of expression of TREM2 in microglia and its specific location, especially in microglial clusters, lead us to speculate that, after FNA, TREM2+ microglia are involved in the phagocytosis of degenerating FMN close to microglial clusters, whereas in the PPT paradigm TREM2 is recognizing myelin debris, that appears at early time-points in this lesion (Jensen et al., 1999). Certain specificity of TREM2 for phagocytosis of myelin debris and apoptotic neurons has already been suggested based also on TREM2 ligands (Gervois and Lambrichts, 2019). In the case of myelin phagocytosis, similarly to our observations, TREM2 increases its expression correlating to myelin debris appearance in demyelinating models (Piccio et al., 2007; Takahashi et al., 2007; Scott-Hewitt et al., 2017). Overexpression of TREM2 in administered bone-marrow-derived cells improved myelin clearance in EAE-induced mice (Takahashi et al., 2005), while TREM2 blockade increased demyelination (Piccio et al., 2007). TREM2KO microglia showed diminished microglia activation and was unable to degrade myelin in cuprizone models (Cantoni et al., 2015; Poliani et al., 2015; Nugent et al., 2020), and accordingly, the treatment with an agonistic antibody against TREM2 enhanced microglial clearance and digestion of myelin, facilitated remyelination and preserved axonal integrity (Cignarella et al., 2020). Our results also suggest that TREM2+ microglia phagocyte myelin debris more efficiently, which may facilitate a rapid lesion resolution after PPT, and the proper axonal collateral sprouting.

On the other hand, a higher avidity in phagocytosis has been established for apoptotic or degenerating cells. *In vitro* studies showed inhibition of phagocytosis of apoptotic neurons after TREM2 knockdown, while on the contrary, apoptotic neurons were cleared more efficiently by TREM2+ BV2 cultured cells (Takahashi et al., 2005; Hsieh et al., 2009). TREM2KO microglia showed also decreased phagocytic capacity, and possibly related to this fact, a reduced age-associated neuron loss was found in TREM2KO aged mice (Linnartz-Gerlach et al., 2019). In our study, TREM2 was mainly expressed in microglial clusters, reported to phagocytose degenerating FMN (Moran and Graeber, 2004), which reinforces the role of TREM2+ cells in neuronal phagocytosis in this model. As reported for PPT, TREM2+ microglia after FNA showed higher phagocytic activity of myelin compared to TREM2- microglia, and suggested that, despite being two different axonal lesions with two different targets, the phagocytic capacity of TREM2+ microglia in both models is independent of their specific lesion particularities.

In line with these results, ApoE, a phagocytic ligand of TREM2, was found in TREM2+ microglia after FNA, but not after PPT. ApoE has been reported to be upregulated after FNA in microglia, as we and other transcriptomic studies found (Tay et al., 2018). Moreover, the ApoE staining in FMN could indicate a role of this molecule as a ligand in TREM2-mediated phagocytosis in clusters, as demonstrated in other studies (Krasemann et al., 2017). Apoptotic neurons can bind to ApoE, and Atagi et al. (2015) demonstrated that its expression enhanced microglial phagocytosis, acting as an opsonin. Consequently, studies on ApoE-KO animals showed decreased FMN death after FNA (Krasemann et al., 2017). To our knowledge, ApoE upregulation has been described in DAM microglia, which has been associated with axonal dystrophy in AD models but is not induced in neuropathic pain (Krasemann et al., 2017; Deczkowska et al., 2018). Given our results, microglial ApoE may not be induced, in specific circumstances, in TREM2+ microglia of injuries affecting only axons, and consequently, TREM2+ microglia could be phagocyting axonal debris after PPT in an ApoE-independent manner.

In this work, we additionally studied another recently described TREM2 ligand, Galectin-3 (Boza-Serrano et al., 2019). In our observations, we found that Galectin-3 was expressed in TREM2+ microglial cells after both types of axonal injuries. Galectin-3 is a molecule extensively related to microglial myelin phagocytosis and clearance (Puigdellivol et al., 2020), which reinforces this role in PPT TREM2+ microglia. Also, after FNA, we observed the expression of Galectin-3 in microglia and on the surface of some FMN, as occurred with ApoE. Galectin-3 is similarly described in the surface of neurons after traumatic brain injury, where it has been suggested to act as an alarmin (Yip et al., 2017; Boza-Serrano et al., 2019) or an opsonizing molecule in the phagocytosis of desialylated neurons (Nomura et al., 2017; Puigdellivol et al., 2020), thus reinforcing this phagocytic role for TREM2+/Galectin-3+ microglia after FNA.

Apart from the phagocytic particularities, TREM2-mediated phagocytosis has been qualified as an “anti-inflammatory,” because it results in the production of anti-inflammatory cytokines (Takahashi et al., 2007; Hsieh et al., 2009) due to an antagonistic role of TREM2 upon NF-κB activation (Yao et al., 2019). Cuprizone studies showed that the absence of TREM2 expression in microglia increases axonal injury and impairs a proper repair (Cantoni et al., 2015; Poliani et al., 2015) that can be rescued with TREM2 activating antibodies (Cignarella et al., 2020). In our study, in both injury models, inflammation is downregulated around the peak of TREM2 expression (Gehrmann et al., 1991; Moran and Graeber, 2004), which indicates a reparatory role for this receptor. As suggested by the above-mentioned studies, the reparatory effect could be the result of the efficient TREM2-mediated phagocytosis accompanied by the secretion of anti-inflammatory cytokines, that could facilitate either axonal sprouting or effective FMN clearance and neuronal regeneration, depending on the model.

Although we centered our attention on microglial TREM2-mediated phagocytosis, it is important to note also that CD16/32+ and CD68+ microglial cells without TREM2 expression were found in both injury models. Our study shows that TREM2 is only triggered in a subpopulation of phagocytic microglial cells and strongly suggests a TREM2-independent microglial phagocytosis. Indeed, some reports indicate that TREM2 is not strictly required for microglia to produce the engulfment of cellular remnants, but it increases the efficient phagocytosis of apoptotic neurons (Hsieh et al., 2009; Cantoni et al., 2015). These results are following our phagocytosis study, where lower percentages of TREM2-independent microglial phagocytosis are observed in all conditions when compared to TREM2+ phagocytosis of myelin. Interestingly, a recent work assigned a role for TREM2 in lipid digestion, excluding its involvement in the uptake of myelin debris during microglial phagocytosis which—according to cell cultures assays performed with TREM2KO bone-marrow-derived cells in this work—may be rescued by high levels of M-CSF (Nugent et al., 2020). Given these results, the relevance of TREM2-mediated phagocytosis may be dependent on the microenvironment in which phagocytosis takes place. On the other hand, the role of sTREM2 in phagocytosis mediated by TREM2- cells may not be excluded. Interestingly, PPT showed increased levels of sTREM2 after lesion, that is not observed after FNA, and are concomitant with high CD68 levels. sTREM2 has been involved in the stimulation of β-amyloid phagocytosis and lysosomal pathways, and it also increased CD68 expression in the AD model (Zhong et al., 2019). Therefore it could be interesting to further explore the role of sTREM2 in the phagocytic function of this model.

Finally, we explored the possibility that TREM2+ cells presented a downregulation of the homeostatic microglial receptor P2RY12. Downregulation of this receptor, together with the expression of TREM2, CD68, CD16/32, Galectin-3, and ApoE has been defined by transcriptomic studies to be associated with the induction of a DAM-phenotype in microglia (Keren-Shaul et al., 2017; Krasemann et al., 2017; Boza-Serrano et al., 2019). In line with these works, our results showed a general P2RY12 downregulation in microglia after PPT in the peak of activation, which is not restricted to TREM2+ microglia. However, after FNA, different patterns of P2RY12 expression were observed in TREM2+ clusters, including clusters with high P2RY12 levels, which suggested the induction of diverse microglial phenotypes within the TREM2+ microglia subpopulation. Similarly, other reports also found activated microglia with high P2RY12 expression in pre-active and chronic white matter lesions of multiple sclerosis and in diffuse β-amyloid plaques in human samples (van Wageningen et al., 2019; Walker et al., 2020). Still, the significance of TREM2 microglia with high P2RY12 levels remain to be explored.

Altogether, the phenotypic characterization of TREM2+ microglia after PPT and FNA indicates a clear involvement of this molecule in a subpopulation of phagocytosing microglia, probably aimed at recognizing myelin debris and degenerating neurons, that could clear more efficiently these cell remnants. In the PPT paradigm, TREM2+ microglia were CD16/32+, CD68+, Galectin 3+, ApoE-, whereas, in the FNA model, TREM2+ microglia in clusters was CD16/32+, CD68+, Galectin-3+, ApoE+ and showed P2RY12 variable expression.

### Pro-inflammatory Rather Than Anti-Inflammatory Local Microenvironments Influence Microglial TREM2 Expression Mainly After PPT but Also After FNA

The final part of this work was to analyze possible modifications of TREM2 in both GFAP-IL6Tg and GFAP-IL10Tg animals after PPT and FNA. To our knowledge, few *in vitro* and *in vivo* data are available on the effect of cytokines on the regulation of microglial TREM2 expression (Petković et al., 2016; Zhai et al., 2017; Zhong et al., 2019). Moreover, our previous studies demonstrated in both Tg animals and after both injury models, differences in microglial activation pattern and injury outcome, which are importantly related to phagocytic.

In the PPT, a higher percentage of microglial cells expressing TREM2 was observed in both GFAP-IL10Tg and GFAP-IL6Tg compared to WT. However, this result did not match with differences in terms of microglial proliferation observed between Tg animals (Recasens et al., 2019), suggesting that other mechanisms related to microglial activation, and possibly related to phagocytosis, are originating differences in delayed axonal sprouting.

In our previous studies in the FNA model, GFAP-IL6Tg showed increased neuronal death and less microglial clusters (Almolda et al., 2014), and GFAP-IL10Tg presented more neuronal survival and microglial clusters (Villacampa et al., 2015) compared to WT. The significant reduction in the TREM2+ area observed in GFAP-IL6Tg mice at 21 dpl could be explained by the lower microglia cluster formation, although our results also indicate that TREM2 expression in microglial clusters is not directly affected by cytokine overproduction, as almost all clusters are TREM2+ in all mouse lines. Indeed, the reduced TREM2 expression in GFAP-IL6Tg due to lower cluster numbers could be explained by a deficient phagocytic capacity in microglia, as already observed in cuprizone-treated GFAP-IL6Tg (Petković et al., 2016). Thus, our results suggest that IL-6 overproduction downregulates TREM2 expression due to changes in the microglial activation pattern, which affect cluster formation, and may not to be a direct effect of IL-6 on TREM2 levels. Moreover, in our study, we did not find a direct link between injury outcome and TREM2 after FNA.

As a whole, TREM2 expression in Tg animals seems to be more influenced by changes in microglial activation pattern that indirectly affects TREM2 expression, than by a direct effect of IL-6 or IL-10 overproduction in phagocytic capacity due to changes in TREM2 microglial expression.

## Conclusions

In conclusion, the results obtained in this work show that TREM2 expression is upregulated after both anterograde and retrograde axonal injury in a specific subpopulation of microglia. Although we have not found a temporal relationship between the induction of proliferation and TREM2 expression in microglial cells, this unique TREM2+ microglia are associated with a more efficient phagocytosis of specific cell remnants, such as myelin and degenerated neurons. However, it is important to highlight that the phenotypic profile of TREM2+ microglial cells in the two models studied present specific characteristics linked to the degree of tissue injury. Concretely, the expression of P2RY12 is only downregulated in microglia after PPT, whereas ApoE expression in TREM2+ cells is exclusively observed in retrograde axotomy, where the axonal injury causes neuronal death. Finally, pro-inflammatory and anti-inflammatory local CNS microenvironments by IL-6 or IL-10 overproduction modified TREM2 expression, mainly after PPT and under pro-inflammatory conditions, due to changes in differential microglial activation but not to a direct cytokine effect.

## Data Availability Statement

The original contributions presented in the study are included in the article/Supplementary Materials, further inquiries can be directed to the corresponding author.

## Ethics Statement

All experimental animal work was conducted according to Spanish regulations (Ley 32/2007, Real Decreto 1201/2005, Ley 9/2003, and Real Decreto 178/2004) in agreement with European Union directives (86/609/CEE, 91/628/CEE, and 92/65/CEE) and was approved by the Ethical Committee of the Autonomous University of Barcelona.

## Author Contributions

GM, AG-L, NV, and MR designed and performed the experiments, including acquiring data and analyzing data; and wrote and revised the manuscript. BA designed experiments, analyzed data; wrote and revised manuscript. KS contributed to design the experiments, analyzed data, and revised the manuscript. BC and BG designed the research and revised the manuscript. All authors contributed to the article and approved the submitted version.

## Conflict of Interest

The authors declare that the research was conducted in the absence of any commercial or financial relationships that could be construed as a potential conflict of interest.

## Supplementary Material

Supplementary Figure 1 Analysis of ApoE expression in astrocytes after PPT. **(A–F)** Representative double-immunolabeled images combining ApoE (green) and GFAP (red) in the NL DG of the hippocampus **(A–C)** and at 2 dpI **(D–F)** after PPT. Note that ApoE expression was easily found on astrocyte-like cells in each condition (white arrows). Scale bar **(A–F)** = 10 μm.

Supplementary Figure 2 Analysis of ApoE expression in astrocytes after FNA. **(A–I)** Representative double-immunolabeled images combining ApoE (green) and GFAP (red) in the ipsilateral NL FN **(A–C)** and at 14 dpI **(D–F)** and 21 dpI **(G–I)**. Note that, in all the time-points, ApoE was detected in astrocytes (white arrows). Scale bar **(A–I)** = 10 μm.

Supplementary Figure 3 Temporal pattern of TREM2 changes in microglial cells in GFAP-IL6Tg and GFAP-IL10Tg after PPT and FNA. **(A–H)** Representative images showing TREM2 staining in the granular (GL) as well as the inner, medial and outer molecular layers (IML, MML, and OML, respectively) of the DG in NL and PPT-lesioned mice at 3, 7, and 21 dpl of GFAP-IL6Tg **(A–D)** and GFAP-IL10Tg mice **(E–H)**. Note that, while in NL TREM2 was only depicted as small rounded morphologies (arrowheads), also found at 21 dpl, at 3 and 7 dpl ramified and occasionally at 21 dpl TREM2+ cells were also observed (arrows). **(I–N)** Representative images showing TREM2 staining in the contralateral NL, as well as the ipsilateral sides of the FN at 14 and 21 dpl of GFAP-IL6Tg **(I–K)** and GFAP-IL10Tg **(L–N)**. In NL TREM2 is mainly restricted to a perinuclear location (arrowheads), whereas at 14 and 21 dpl TREM2 is extended to microglia ramifications and clusters (arrows). Scale bar **(A–H)** = 50 μm; **(I–N)** = 30 μm.
