# Supplementary figures and images for: Differential Roles of TREM2+ Microglia in Anterograde and Retrograde Axonal Injury Models
Source: Front Cell Neurosci. 2020 Nov 20;14:567404. doi: 10.3389/fncel.2020.567404 (PMC7715005; doi:10.3389/fncel.2020.567404)

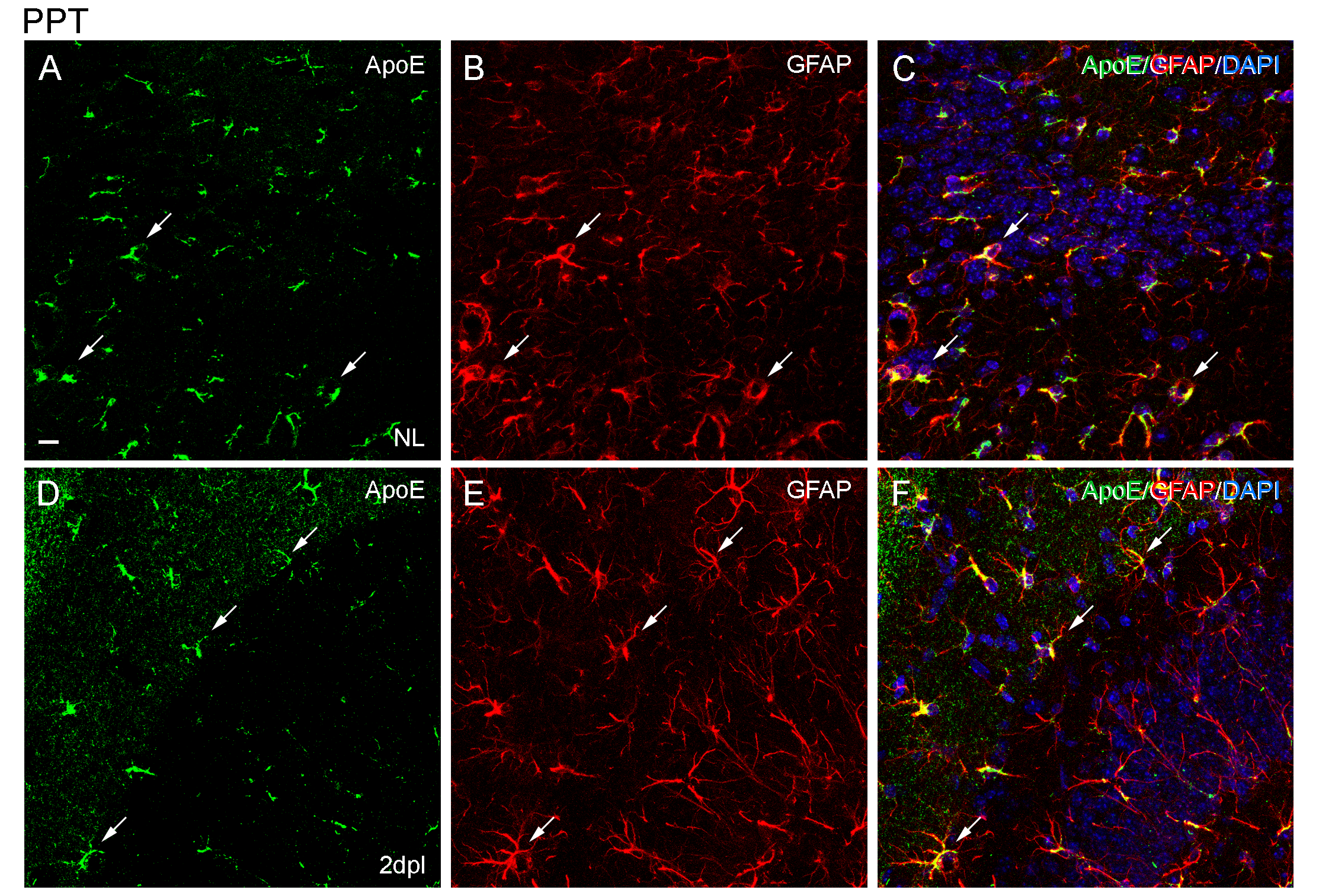

Supplement: Supplementary Figure 1 — Analysis of ApoE expression in astrocytes after PPT. (A–F) Representative double-immunolabeled images combining ApoE (green) and GFAP (red) in the NL DG of the hippocampus (A–C) and at 2 dpI (D–F) after PPT. Note that ApoE expression was easily found on astrocyte-like cells in each condition (white arrows). Scale bar (A–F) = 10 μm. [file Image_1.tif]

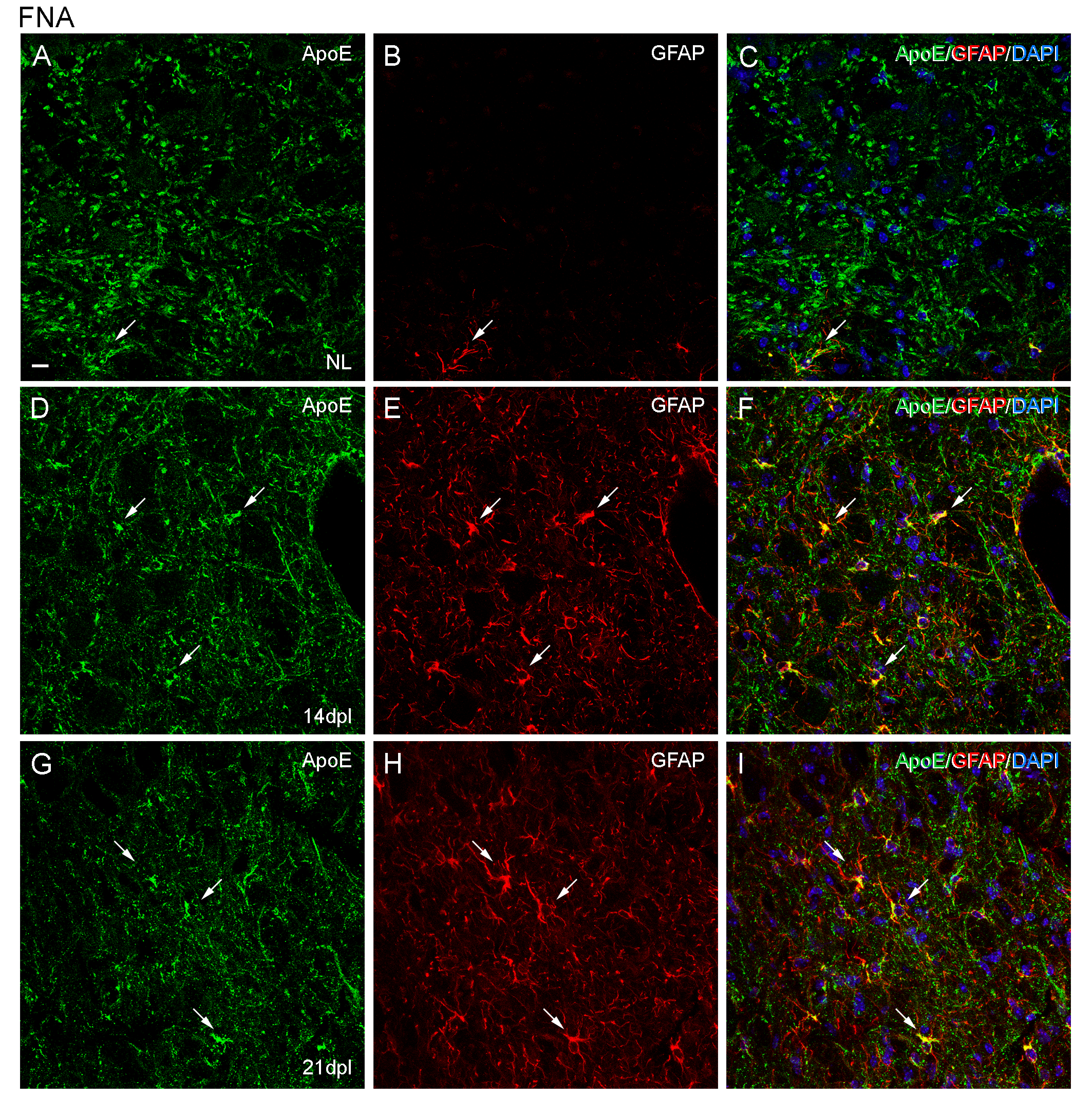

Supplement: Supplementary Figure 2 — Analysis of ApoE expression in astrocytes after FNA. (A–I) Representative double-immunolabeled images combining ApoE (green) and GFAP (red) in the ipsilateral NL FN (A–C) and at 14 dpI (D–F) and 21 dpI (G–I). Note that, in all the time-points, ApoE was detected in astrocytes (white arrows). Scale bar (A–I) = 10 μm. [file Image_2.tif]

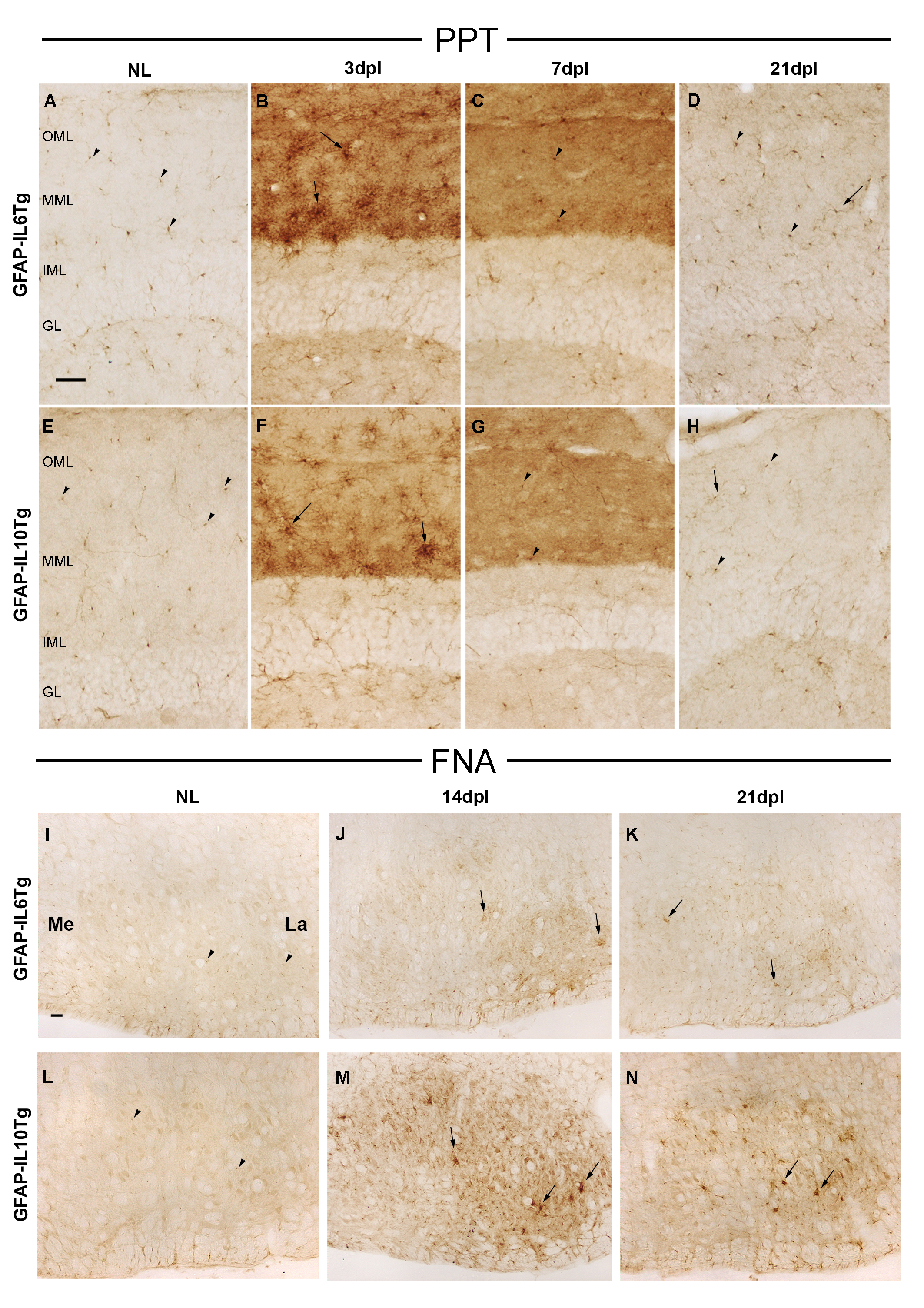

Supplement: Supplementary Figure 3 — Temporal pattern of TREM2 changes in microglial cells in GFAP-IL6Tg and GFAP-IL10Tg after PPT and FNA. (A–H) Representative images showing TREM2 staining in the granular (GL) as well as the inner, medial and outer molecular layers (IML, MML, and OML, respectively) of the DG in NL and PPT-lesioned mice at 3, 7, and 21 dpl of GFAP-IL6Tg (A–D) and GFAP-IL10Tg mice (E–H). Note that, while in NL TREM2 was only depicted as small rounded morphologies (arrowheads), also found at 21 dpl, at 3 and 7 dpl ramified and occasionally at 21 dpl TREM2+ cells were also observed (arrows). (I–N) Representative images showing TREM2 staining in the contralateral NL, as well as the ipsilateral sides of the FN at 14 and 21 dpl of GFAP-IL6Tg (I–K) and GFAP-IL10Tg (L–N). In NL TREM2 is mainly restricted to a perinuclear location (arrowheads), whereas at 14 and 21 dpl TREM2 is extended to microglia ramifications and clusters (arrows). Scale bar (A–H) = 50 μm; (I–N) = 30 μm. [file Image_3.tif]
